# Electrocaloric Effect on Lead-Free Ferroelectrics: Challenges in Identifying Trends and Evaluating Predictive Models

**DOI:** 10.3390/ma18194444

**Published:** 2025-09-23

**Authors:** Magdalena Krupska-Klimczak, Michał Frontczak, Zdobysław Świerczyński, Serhii Semenov, Dariusz Kajewski, Irena Jankowska-Sumara

**Affiliations:** 1 Institute of Security and Computer Science, University of National Education Commission, Podchorążych 2, 30-084 Kraków, Poland; michal.frontczak1@uken.krakow.pl (M.F.); serhii.semenov@uken.krakow.pl (S.S.); 2 Faculty of Exact and Natural Sciences, University of National Education Commission, Podchorążych 2, 30-084 Kraków, Poland; irena.jankowska-sumara@uken.krakow.pl; 3 Institute of Physics, University of Silesia in Katowice, ul. 75 Pułku Piechoty 1, 41-500 Chorzów, Poland; dariusz.kajewski@us.edu.pl

**Keywords:** electrocaloric effect, lead-free ceramics, machine learning

## Abstract

The electrocaloric effect (ECE) has become one of the most intensively studied topics in ferroelectrics, with dozens of new papers that report experimental results and provide increasingly extensive data compilations every year. However, the heterogeneity of the literature, arising from differences in compositions, dopants, preparation routes, measurement protocols, and analysis methods, makes direct comparison between studies highly problematic. In this work, we focus on barium titanate (BaTiO_3_) as a representative lead-free ferroelectric system. BaTiO_3_ was chosen because, within this class of materials, it offers by far the largest body of reported ECE results, obtained under a wide range of experimental conditions, thus allowing for the most comprehensive characterization. Using this example, we explore whether meaningful patterns related to the influence of chemical substitution on the magnitude and temperature dependence of the ECE can be discerned. In addition, we critically examine why certain comparisons reported in the literature may be misleading or inherently unreliable. Finally, we discuss predictive approaches, including those employing artificial intelligence algorithms, and evaluate their applicability and limitations in modeling the electrocaloric response.

## 1. Introduction

The electrocaloric effect (ECE) has attracted significant interest over recent decades due to its potential for enabling efficient, environmentally friendly solid-state cooling technologies [1]. This phenomenon, which involves reversible temperature changes in certain materials upon application or removal of an electric field, is seen by many researchers as a promising alternative to traditional refrigeration methods that rely on greenhouse gases [2]. However, despite the enthusiasm surrounding ECE, the field remains controversial. On one hand, the vast potential of ECE is widely acknowledged, with numerous reports highlighting large temperature changes in specific materials under certain conditions [3]. On the other hand, no practical electrocaloric (EC) material or device has yet been developed that consistently delivers the performance required for widespread technological adoption [4]. This contradiction underscores the complexities inherent in understanding and harnessing the ECE.

Initial research efforts into ECE largely focused on individual experimental measurements conducted on select materials, often under narrowly defined conditions [5,6]. These studies provided valuable, detailed insights into the fundamental properties and behaviors of candidate EC materials, contributing to a growing but fragmented knowledge base. However, isolated data points alone are insufficient to comprehend the underlying mechanisms governing ECE fully or to guide the rational design of improved materials. As research in this field expanded, numerous experimental results accumulated in the literature, spanning different material compositions, processing methods, and measurement techniques [1,7,8]. Such a wealth of data should, in principle, facilitate the identification of consistent trends and empirical relationships critical for advancing ECE technology.

Yet, the reality is more complicated. The literature on EC materials is characterized by considerable variability and a lack of systematic organization. Different synthesis and experimental protocols, diverse material systems, and inconsistent reporting standards contribute to a highly heterogeneous dataset. This absence of uniformity makes it challenging to extract clear correlations or to construct predictive models based purely on traditional data analysis. In particular, studies on barium titanate BaTiO_3_-based ceramics, which are among the most extensively researched EC materials, exemplify this issue. Results for BaTiO_3_ ceramics display significant scatter, with reported values for key EC parameters differing widely depending on subtle variations in material composition, processing, and measurement methodology [9,10,11].

This highlights a pressing need for systematic data acquisition, standardization, and curation tools to aggregate and harmonize the available data, enabling more reliable analysis and trend discovery.

The intrinsic complexity of the ECE itself further compounds the challenges in data handling. Predicting the magnitude and behavior of ECE in a given material system requires understanding a confluence of factors, including crystal structure, phase transitions, defect chemistry, and electric field interactions, all of which can vary in subtle and nonlinear ways. As a result, conventional theoretical and empirical approaches often fall short in delivering accurate, generalizable predictions. In this context, modern data-driven techniques such as machine learning (ML) have emerged as promising alternatives. Machine learning algorithms can uncover hidden patterns within complex datasets and predict EC performance based on a range of material descriptors. Several recent publications have demonstrated the feasibility of applying ML models to ECE data [12,13,14,15], offering new pathways for accelerated materials discovery and optimization.

Nonetheless, despite the promise of machine learning, significant limitations remain. The scarcity of large, high-quality, consistent datasets restricts robust models’ training and validation [16]. Moreover, many ML approaches currently function as “black boxes,” providing limited physical interpretability of their predictions, which hinders their acceptance and utility within the materials science community [17]. These drawbacks point to the necessity of integrating domain knowledge with advanced data analytics and ensuring rigorous data collection protocols. Furthermore, practical deployment of ML-based predictive tools requires careful consideration of model uncertainty, transferability, and scalability [18,19].

In this context, the present work is structured to provide a comprehensive and critical overview of the current state of research on the ECE. We begin by summarizing the principal experimental methodologies employed for measuring ECE, highlighting their strengths and inherent limitations. This discussion serves as a foundation for evaluating the reliability and comparability of published results. Building upon this, we present a focused analysis of literature data concerning BaTiO_3_-based ceramics, encompassing both undoped and doped compositions [20,21,22,23,24,25,26,27,28,29,30,31,32,33,34,35,36,37,38,39,40,41,42,43,44,45,46,47,48,49,50,51,52,53,54,55,56,57,58,59,60,61,62,63,64,65,66,67,68,69,70,71,72,73,74,75,76]. By systematically compiling and organizing these data, we aim to identify possible trends and correlations that may shed light on the factors governing EC performance. At the same time, we critically assess whether such an effort is justified in view of the significant variability and lack of standardization in the literature. Finally, we review the predictive approaches available in the current body of research, with particular emphasis on machine learning–driven strategies. By evaluating both their achievements and shortcomings, we aim to delineate the prospects and limitations of prediction tools as a step toward more rational and reliable design of EC materials.

## 2. Materials and Methods

### 2.1. Measurement Techniques of the Electrocaloric Effect

The ECE can be determined by two main methods: direct measurements, in which the actual change in sample temperature or heat transfer is recorded upon application of an electric field, and indirect methods, in which the effect is calculated from other measured quantities using the laws of thermodynamics [77,78,79]. Due to experimental difficulties, i.e., the lack of commercial measurement systems for direct characterization of ECE and the need to meet stringent conditions (e.g., adiabatic), in practice, most experimental data come from indirect methods as will be shown later in this article.

#### 2.1.1. Formalism Based on Maxwell’s Relations

**Figure 1:**
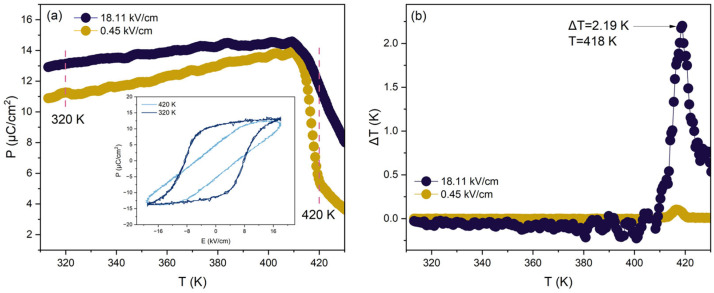
Example of the indirect method for determining the electrocaloric effect: (**a**) temperature dependence of polarization with inset hysteresis loops, and (**b**) the corresponding ΔT calculated using Maxwell’s relations.

The indirect method based on Maxwell’s relations determines the entropy change in the system from measurements of polarization as a function of applied electric field and temperature (Figure 1a) and subsequently calculates the ECE using thermodynamic relations [77] (Figure 1b). This method relies on the Maxwell relation for thermodynamic systems under an electric field, which links the entropy change (∆*S*) to the change in electric field intensity (∆*E*) and the polarization change (∆*P*) to the temperature change (∆*T*) of the material:

$${\left(\frac{\partial S}{\partial E}\right)}_{T}={\left(\frac{\partial P}{\partial T}\right)}_{E}$$

As a result of integration of this dependence, the isothermal change in entropy ∆*S* caused by the change in field from *E*_1_ to *E*_2_ can be determined:

$$\Delta S\left(E_{1}\to E_{2},T\right)=\int_{E_{1}}^{E_{2}}{\left(\frac{\partial P}{\partial T}\right)}_{E}dE\;$$

In turn, the expected adiabatic temperature change ∆*T*_ad_ related to the ECE (assuming no heat exchange with the environment) is:

$$\Delta T_{ad}=-\frac{T}{\rho C}\int_{E_{1}}^{E_{2}}{\left(\frac{\partial P}{\partial T}\right)}_{E}dE$$

The above formula results from the fact that ∆S corresponds to the entropy change in an isothermal process, while in adiabatic conditions, this entropy change results in an opposite temperature change (negative sign, because an increase in entropy causes a decrease in ∆*T* under adiabatic conditions). In the above equations, *C* denotes the specific heat (at constant pressure, or possibly at a constant field *E*) of the material, and *ρ* is the sample density. It is usually assumed that C does not depend strongly on the electric field and its values measured at *E* = 0 can be used (unless a sharp phase transition occurs). However, the change in heat capacity with temperature *C* = *C*(*T*) within the measurement range is typically taken into account.

In practice, the implementation of the Maxwell formalism requires measuring the polarization hysteresis loop *P*(*E*) as a function of temperature. Typically, a series of *P*(*E*) measurements is made at a series of constant temperatures, uniformly covering the range of interest (e.g., around the phase transition temperature of the material, where the effect is strongest). Hysteresis loop measurements are performed under quasi-static conditions, with slow field changes, often on the order of 10^−1^–10^0^ Hz, so that the sample remains close to thermodynamic equilibrium and the Maxwell relation is applicable (irreversible effects related to energy dissipation are neglected) [80,81]. The sample must be equipped with electrodes and placed in a temperature-stabilized measurement chamber (e.g., a thermostatic oven or a cryostat) with accurate temperature control and measurement. In addition to the hysteresis loop, the specific heat of the material must be determined (for the calculation of ∆*T*_ad_). Usually, separate measurements (e.g., DSC) or literature data for the tested substance are used.

The Maxwell approach is very popular due to its experimental accessibility. A standard hysteresis loop measurement setup is sufficient to determine the effect, without the need for direct detection of thermal signals. Most published ECE results were obtained using this method, integrating *P*(*T*,*E*) data. This method allows for the determination of both the isothermal entropy change ∆*S* and the corresponding adiabatic temperature change ∆*T*_ad_ over a wide range of temperatures and fields. The limitations are essentially the temperature range of the measuring equipment and the maximum field strength that can be applied to the sample (limited by the electrical strength of the material). However, it should be emphasized that the computational nature of the Maxwell method poses certain limitations and potential sources of error. First of all, Maxwell’s relation is strictly valid only for reversible and quasi-static processes. If significant hysteresis, dielectric losses, or frequency dependence are present in the polarization, the resulting calculated ∆*T*_ad_ may deviate from reality. In particular, in relaxor-type materials, where local inhomogeneities occur (so-called polar nanoregions) and a strong dependence on the field application rate, Maxwell’s method may lead to significant discrepancies with direct measurements. For example, it has been found that in such cases the indirectly calculated temperature change can even be of the opposite sign (so-called apparent negative electrocaloric effect), while accurate direct measurements show only a positive effect [82]. The uncertainty of Maxwell’s method results from the cumulation of the uncertainty of the polarization and temperature measurements, as well as from the procedure of differentiation and integration of the data—the discrete and often limited dataset *P*(*T*) requires approximation (e.g., function fitting) and numerical differentiation, which may introduce errors. Furthermore, the accuracy of the estimate of ∆*T*_ad_ depends on the precision of the knowledge of the specific heat *C*; any uncertainty or neglect (e.g., neglecting changes in *C*(*T*)) linearly affects the calculated ∆*T*_ad_. In summary, the Maxwell formalism is a powerful analytical tool for ECE evaluation, simple to implement and widely used, but it requires careful data acquisition and may fail in situations deviating from the thermodynamic equilibrium of the sample.

The ECE can also be evaluated by applying the indirect Maxwell relation to pyroelectric measurements [83,84]. In this approach, the key quantity is the temperature derivative of the polarization at constant electric field, which corresponds to the pyroelectric coefficient. Experimentally, this coefficient is obtained by monitoring the pyroelectric current during a controlled temperature sweep under a fixed applied field. If the sample has surface area *A* and the heating or cooling rate is $\beta =\frac{dT}{dt}$, the current $I_{p}$ is related to the pyroelectric coefficient through $p\left(T,E\right)=\frac{I_{p}\left(T,E\right)}{\left(A\beta \right)}$. Measuring this current as a function of temperature and field thus yields the desired ${\left(\frac{\partial P}{\partial T}\right)}_{E}$.

Once the pyroelectric coefficient has been determined, it can be introduced into the Maxwell relation linking entropy and polarization. For an adiabatic change in the electric field from zero to *E*, the EC temperature change is calculated according to:

$$\Delta T\left(T,E\right)=-\frac{T}{\rho c}\int_{0}^{E}p\left(T,E\prime \right)dE\prime ,$$

where *ρ* and *c* are the density and specific heat of the material, respectively. In practice, the integral over field is evaluated numerically using the set of pyroelectric data recorded at different bias fields, and the temperature dependence of the heat capacity is either measured independently or taken from literature values.

It must be emphasized, however, that the accuracy of this approach is affected by the complexity of the pyroelectric response itself. The measured pyroelectric coefficient generally includes both a primary contribution, which reflects the intrinsic change in spontaneous polarization with temperature at constant strain, and a secondary contribution, which originates from thermally induced strains coupled through the piezoelectric effect. Because it is experimentally challenging to separate these two terms, the pyroelectric data used in the Maxwell relation may not represent the purely intrinsic polarization change. As a consequence, the EC temperature change derived from such measurements can be overestimated or underestimated depending on the magnitude and sign of the secondary contribution. This limitation should be kept in mind when interpreting EC results obtained by the pyroelectric indirect method.

#### 2.1.2. Differential Scanning Calorimetry (DSC)

Differential scanning calorimetry (DSC) is a direct thermal measurement method, but it can be considered an indirect technique for determining the ECE, as it provides information on the entropy change based on the measured heat flow [85]. DSC involves the precise measurement of the heat flux difference between a sample and a reference material during a controlled temperature program. In the context of ECE, modified commercial DSC devices are used, allowing for the application of a high voltage to the sample during the measurement. This allows for direct measurement of the heat exchanged by the EC material when the electric field changes, and then the corresponding entropy change ∆*S* and (if C is known) the adiabatic temperature change ∆*T*_ad_ can be determined [77,79]. In other words, DSC records the thermal effect accompanying the polarization or depolarization of the sample. If the measurement is performed under isothermal conditions (ambient/sample temperature maintained constant), the released or absorbed heat Q related to the ECE corresponds to the entropy change ∆*S* = *Q*/*T*. In turn, if the sample changes temperature (e.g., in almost adiabatic conditions inside the DSC), the recorded temperature curve can be used to calculate ∆*T*_ad_ directly. Most often, however, the isothermal or quasi-isothermal mode is used: the sample is kept at a constant temperature and at some point, the electric field is turned on (or off), observing in the DSC thermogram, respectively, an exothermic peak (heat release upon material polarization) or an endothermic peak (heat absorption upon depolarization). The equivalence of the areas of both peaks (of opposite sign) proves the reversibility of the effect and the absence of irreversible losses [77,80]. On this basis, the value of the exchanged heat can be calculated, and hence ∆*S*. Alternatively, for materials exhibiting phase transitions, constant-field scanning measurements are performed: the sample is heated or cooled at a given rate under various constant field strengths *E* (so-called isofield measurements), and DSC curves are recorded. A set of such curves reveals the shift in the phase transition temperature under the influence of the field (e.g., the shift *T*_C_) and the change in the enthalpy of the transition. Using the Clausius–Clapeyron equation or direct integration of heat flux differences, the entropy and temperature change associated with the EC effect can be determined.

The advantage of DSC is its high sensitivity and precision in detecting enthalpy changes. Modern differential calorimeters can detect thermal effects on the order of microjoules, enabling the observation of even very small ECE [77,78]. Furthermore, DSC records the full thermodynamic response of the sample, including both reversible effects such as ECE and potential side processes (e.g., unwanted Joule’s losses), allowing them to be distinguished and corrected. The literature emphasizes that DSC is a more accurate alternative to simpler heat measurements (e.g., using thermocouples). Ensuring stable conditions and a controlled temperature profile in DSC minimizes noise and random errors; typical measurement repeatability is very good, and calorimetric signals at the level of a few milliwatts are easily distinguished from the background. Furthermore, DSC allows for continuous study of the EC effect as a function of temperature (by recording the signal during temperature scanning), which provides rich information, for example, about the temperature range in which the effect occurs or the complexity of phase transitions.

The limitations and challenges of the DSC method include the need to modify the apparatus. Special measurement cells with high voltage feed-throughs are required, which present technical difficulties and can restrict the available field range (typically to several dozen kV/cm) [78]. The sample must be small (usually several dozen mg or less) and properly prepared: it is placed in a measuring crucible, often as a thin plate or layer with integrated electrodes. Avoiding measurement artifacts caused by Joule’s heating is essential. If the tested material has finite electrical conductivity or substantial dielectric losses, then the current passing through the sample during field application generates additional heat, which overlaps with the EC signal. In a well-conducted experiment, leakage currents should be kept below 10^−8^ A so that resistive heat in the sample remains negligible. This is practically verified by observing the DSC signal: after switching off the field, it should return to the baseline, indicating no further sample heating. Otherwise, it is necessary to improve sample insulation or adjust for losses. Another limitation is the relatively slow rate at which the electric field can be changed in DSC. Although the field usually changes abruptly, the measurement itself has limited temporal resolution (on the order of seconds). Therefore, this technique records the equilibrium or slowly varying thermal response; it does not capture the ultrafast dynamics of the effect (for such purposes, thermal imaging with an infrared camera with high sampling rate is employed). The uncertainty of DSC results mainly stems from errors in heat flux calibration and background subtraction. The baseline must be determined with high precision (for example, by performing a separate measurement with the sample absent an electric field) and the field signal subtracted to extract the pure ECE. Small variations in the sample’s heat capacity or minor thermal drifts can lead to an uncertainty in the calculated ∆*S* of several percent. Nonetheless, a correctly performed DSC measurement offers very reliable quantitative ECE results, often serving as a benchmark for verifying indirect Maxwell-based methods.

In summary, DSC enables the direct determination of the ECE by measuring heat transfer. Typical experiments are performed at temperatures within the range of interest for applications (e.g., near room temperature for cooling materials) using an electric field of several to several tens of kV/cm. Experimental conditions are selected to ensure near reversibility of the process (low leakage currents, repeatable on/off cycles) and good thermal signal resolution (slower temperature scanning improves enthalpy’s resolution at the expense of measurement time).

#### 2.1.3. Heat Flux Measurement Methods (Direct Calorimetry)

The third group of methods consists of techniques involving the direct measurement of the heat flux released or absorbed by the sample during polarization changes. Unlike DSC, these methods often use simpler calorimetric systems designed specifically for EC measurements. An example is quasi-adiabatic calorimetry with thermocouples. In this configuration, the EC material is thermally isolated (placed in a container with very good thermal insulation), and one or more thermocouples are placed near the sample to measure the temperature difference between the sample and the environment [8]. When an external electric field is applied (or is disconnected), the dipoles in the material become ordered (or deordered), resulting in the release or absorption of a certain amount of heat. Under ideal adiabatic conditions, this would cause a change in the sample temperature ∆*T*. However, in practice, some heat flows to the environment—and it is this heat flux that is detected by thermoelectric sensors. The signal from a thermocouple (or a system of thermocouples connected in series to increase sensitivity, so-called thermopile) allows for the determination of a small temperature difference ∆*T* between the sample and the housing, proportional to the heat flow. By measuring this signal as a function of time, the total exchanged energy *Q* can be determined by integrating the signal (e.g., thermoelectric voltage) over the duration of the effect. Then, similarly to the DSC method, the entropy change ∆*S* = *Q*/*T* (assuming a known mean process temperature) and the adiabatic temperature change

$$\Delta T_{ad}=-\frac{T\Delta S}{C}$$

(taking into account the sample’s heat capacity) are calculated. Alternatively, if the measuring system is sensitive enough to detect only the sample’s ∆*T* (with minimized heat loss), this result directly reflects the ECE.

Heat flux measurement methods are characterized by simpler equipment than DSC. Often, the construction of such a calorimeter involves shielding the sample with an insulating material and installing temperature sensors, which can be achieved relatively easily even in laboratory conditions. This allows for the study of samples of various sizes, including larger ones than in DSC (limited by the crucible size and the required low mass to avoid signal attenuation). Furthermore, these systems allow for faster electrical field changes—it is possible to study the pulsed action of the electric field and the dynamic thermal response of the material [86,87]. They are often used to test prototype cooling elements, where the efficiency of heat transfer during cyclic polarization of the material is important. In the literature, such methods are also referred to as quasi-direct methods because they measure heat (or temperature) directly but usually require some interpretation and intermediate calculations to obtain ∆*T*_ad_.

The disadvantages and limitations of this group of methods include lower accuracy and resolution compared to professional DSCs. Signals from thermocouples (in the microvolt range) require amplification and careful calibration. The achieved sensitivity is limited. The minimum detectable ∆*T* is usually several milliKelvins, while the best DSCs can indirectly detect fractions of a milliKelvin corresponding to the thermal effect. Furthermore, it is difficult to achieve perfectly adiabatic conditions: there are always small heat losses to the environment, which may result in a slightly underestimated measured signal. In practice, calorimetric corrections are introduced. For example, a calibration procedure involves generating a known amount of heat within the system (e.g., by a Joule current pulse across a built-in resistor) and measuring the sensor response. Based on this, the effective time constant of the system and the proportionality of the signal are determined. It has been reported that corrections of 20–30% are necessary in typical experiments to compensate for thermal losses and imperfect insulation [77,80]. Despite these difficulties, once the system is calibrated, reproducible quantitative results can be obtained [86]. It should also be noted that thermocouple methods integrate the signal across the entire sample, making it more difficult to distinguish local effects or material inhomogeneities, unlike, for example, thermal imaging cameras, which depict the distribution of ∆T across the sample surface.

Typical experimental conditions for heat flux measurement methods involve maintaining the sample at a constant ambient temperature (often room temperature or another temperature of interest) and suddenly applying or removing an electric field with a defined amplitude. A short-duration thermal signal is recorded (the thermocouple voltage peak) lasting from fractions of a second to several seconds, depending on the rate of heat exchange with the environment. The sample is often placed in a vacuum or an inert gas atmosphere to eliminate convection and moisture (which could affect the signal). The electric field used is often similar to that used in other methods (on the order of several hundred kV/m); however, ensuring good electrical insulation within the calorimeter is crucial to avoid current leakage through the thermocouples or the chamber walls. These measurements are typically performed at a single temperature, possibly repeated at different sample temperatures to determine the ∆*T*_ad_(*T*) curve, which, however, requires time-consuming stabilization of the sample at the next temperature level.

In terms of uncertainty, the dominant bias is the systematic error associated with the non-ideal adiabatic nature of the system. If some heat escapes to the environment before it is measured, the resulting ∆*S* will be underestimated. Therefore, the aforementioned calibration is crucial. Additionally, there are random errors in thermocouple voltage measurement (electronic noise, amplifier zero drift, etc.). The total uncertainty of the obtained ∆*T*_ad_ is usually greater than in the DSC method, estimated at several percent. Nevertheless, heat flux measurement methods are a valuable complement to ECE studies, especially at the stage of prototype cooling devices, where obtaining a direct thermal signal from the material is crucial.

In summary, the indirect method based on Maxwell’s equations provides quick and relatively simple estimates but is prone to systematic errors and is predictive in nature. DSC offers high sensitivity and provides rich information about phase transitions, but its results do not directly reflect the actual adiabatic Δ*T*. The heat flux method, on the other hand, allows for the observation of the effect under dynamic conditions, but at the cost of lower sensitivity and the need for precise thermal calibration. In practice, the best results are obtained by combining these methods: theoretical predictions based on Maxwell’s relations allow for the expected range of the effect, DSC enables analysis of the thermodynamic mechanisms of the phenomenon, and heat flux measurements bring the experiment closer to the actual application conditions. The combined use of these techniques allows for both quantitative and qualitative characterization of the ECE in a given material.

### 2.2. Literature Data Collection

To provide a consistent foundation for trend analysis, we systematically collected EC data for BaTiO_3_-based ceramics from the available literature and presented it in the Appendix A. The search used major scientific databases such as Web of Science and Scopus, with keywords including “electrocaloric effect”, “BaTiO_3_ ceramics”, and “doped BaTiO_3_”.

The dataset was deliberately restricted to BaTiO_3_-based ceramics, including both undoped and doped compositions, while excluding single crystals, thin films, and other forms. This choice was motivated by several factors. BaTiO_3_ is among the most extensively studied ferroelectric materials, providing a sufficiently large body of high-quality experimental data to enable meaningful statistical analysis. Furthermore, its relatively low Curie temperature (T_C_), compared to other ferroelectrics such as PZT, allows for EC measurements to be conducted under more accessible laboratory conditions. The ceramic form was specifically selected due to its technological relevance and the well-established processing methods, which reduce variability associated with microstructural differences that are more pronounced in single crystals or thin films. Focusing on ceramics thus ensures a consistent material platform for trend analysis and provides a reliable foundation for developing predictive models of the ECE.

For each selected study, quantitative EC data were extracted directly from tables provided by the authors or, when tables were unavailable, approximated from published graphs. In the latter case, the maximum Δ*T* reported was identified, and its corresponding temperature and electric field were recorded. Whenever possible, the measurement methodology (direct or indirect) was also noted, as this distinction is essential for evaluating the comparability and reliability of reported results. This dual approach allowed us to include well-tabulated datasets and works where results were only graphically represented, thereby maximizing the coverage of the literature.

For each entry, information was organized in a standardized tabular format comprising measurement temperature *T*, EC temperature change Δ*T*, applied electric field *E*, EC strength Δ*T*/*E*, measurement technique (direct or indirect), and literature reference. An example of this organization is presented in Table 1. This structured approach enabled subsequent visualization and analysis of potential trends across different studies.

It should be emphasized, however, that the extracted dataset remains affected by the intrinsic heterogeneity of the literature. Variability in measurement protocols, reporting standards, and data presentation introduces unavoidable uncertainties, particularly in cases where values were digitized from figures rather than obtained directly from numerical tables. As such, the resulting compilation should be regarded as a representative but non-exhaustive dataset, suitable for identifying general tendencies rather than providing definitive quantitative benchmarks. This aspect will be discussed in more detail in Section 4.

**Figure 2:**
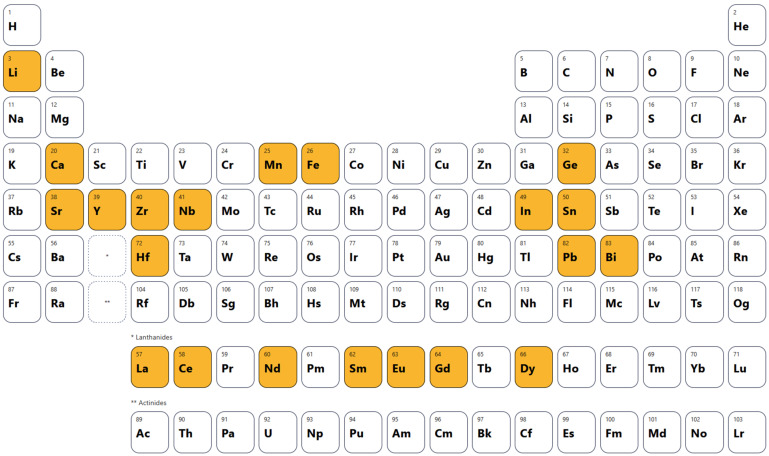
Elements reported in the literature as dopants in BaTiO_3_, highlighted on the periodic table.

The dopants identified across the surveyed studies span a broad range of the periodic table (Figure 2), reflecting the diverse strategies employed to tailor the EC response of BaTiO_3_-based ceramics. Substitutions at the A-site, such as Ca, Sr, and La, are commonly used to adjust the lattice constant and shift the Curie temperature (T_C_), optimizing the working temperature window [88,89,90].

At the B-site, elements including Zr [91,92], Hf [93], Nb [94], Mn [95], Fe [96], Ge [97], and In [98] play a crucial role in modifying polarization behavior, broadening phase transitions, or influencing electrical conductivity through donor–acceptor effects. Heavy p-block elements such as Bi [99] and Pb [34] introduce strong polarizability due to their lone-pair electrons, often enhancing ferroelectric distortions. Meanwhile, rare-earth dopants (La, Ce, Nd, Sm, Eu, Gd, Dy) are frequently employed to fine-tune Tc, suppress leakage currents, and stabilize the microstructure [100]. The wide chemical diversity of dopants highlights the absence of a single universal strategy; instead, different substitutions are chosen depending on whether the primary aim is to shift transition temperatures, maximize ΔT, improve dielectric stability, or reduce losses under high electric fields. It should be emphasized, however, that at this stage the overview serves only to illustrate the compositional landscape of the studies included in this review. At the same time, the impact of specific dopants on the magnitude of Δ*T* will be analyzed and discussed in detail in Section 3.1.

**Figure 3:**
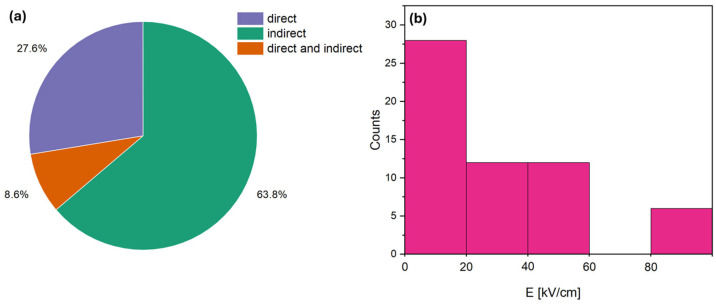
(**a**) Distribution of EC measurements according to the applied method (indirect, direct, or combined). (**b**) Histogram of electric field strengths employed in the reported studies.

The analysis of the collected dataset indicates a clear predominance of a single measurement approach: among the 58 studies considered, 37 employed the indirect method, 16 the direct method, and only 5 utilized both techniques concurrently (Figure 3). This disparity may be attributed to practical considerations, as the indirect method is generally easier to implement and provides rapid estimates of the ECE. However, it should be noted that indirect measurements represent approximations, as the effect is calculated rather than directly observed. In contrast, direct measurements yield the actual EC response but necessitate specialized equipment and more rigorous experimental conditions, limiting their widespread adoption.

Importantly, the concurrent application of both methods on the same samples would allow for a more robust evaluation of the discrepancies between calculated and measured values. Such data are critical for developing reliable machine-learning-based predictive models, as they enable calibration against actual material behavior and reduce systematic uncertainties inherent to either method individually. Therefore, while the assembled dataset facilitates identification of general trends in BaTiO_3_-based ceramics, its heterogeneity and the limited use of dual-method measurements constitute a significant constraint on constructing accurate and generalizable predictive tools.

Regarding the magnitude of the applied electric field, expressed in kV/cm, a majority of the studies investigated relatively low fields: in 28 of the 58 studies, the field was below 20 kV/cm, in 12 studies it ranged from 21 to 40 kV/cm, in another 12 studies from 41 to 60 kV/cm, and only 6 studies applied fields above 80 kV/cm, with no studies reported in the intermediate range of 61–80 kV/cm. Studies applying very high fields, exceeding 100 kV/cm, remain scarce due to the significant experimental challenges they pose. At such field strengths, the risk of dielectric breakdown, sample degradation, and irreversible changes in microstructure increases, limiting the reproducibility and reliability of the measured EC response. Maintaining uniform fields across ceramic samples also requires careful electrode design and precise control of sample geometry. From an application perspective, the predominance of lower fields reflects technologically accessible and safe operating conditions, as high fields are generally impractical for solid-state cooling devices due to safety concerns, increased energy consumption, and reduced long-term reliability. Nevertheless, data obtained at very high fields are valuable for fundamental studies and calibrating predictive models, as they provide insights into the ultimate EC performance limits of BaTiO_3_-based ceramics.

## 3. Results

### 3.1. Literature Overview of Electrocaloric Effect in BaTiO_3_-Based Materials

**Figure 4:**
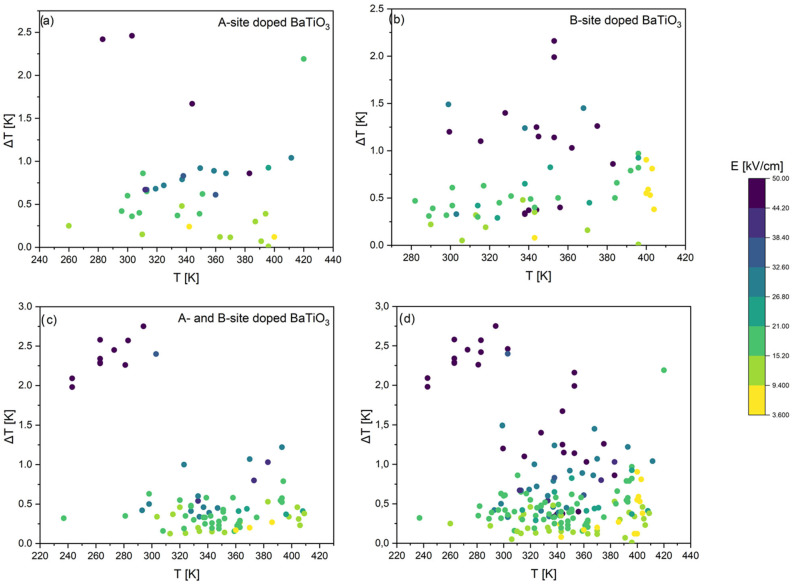
Comparative electrocaloric temperature change (Δ*T*) as a function of measurement temperature for BaTiO_3_ ceramics: (**a**) A-site doped, (**b**) B-site doped, (**c**) co-doped at A- and B-sites, and (**d**) all compositions combined [20,21,22,23,24,25,26,28,29,30,31,33,34,36,37,38,39,40,41,42,43,44,45,46,47,48,50,51,52,53,54,55,56,57,58,59,60,61,62,63,64,65,66,67,68,70,71,72,73,74,75,76]. Color scale indicates the applied electric field strength; only data obtained below 50 kV/cm are included.

The comparative plots presented in Figure 4 provide an overview of the ECE in BaTiO_3_-based materials, grouped according to the type of dopant. Panels (a)–(c) separate the results into A-site dopants, B-site dopants, and combined A- and B-site co-doping, while panel (d) compiles all available data into a single diagram. The vertical axis represents the EC temperature change Δ*T*, while the horizontal axis corresponds to the measurement temperature *T*. The magnitude of the applied electric field E is indicated by the color scale, with darker shades representing stronger fields. In this comparative analysis, the dataset is limited to results obtained under electric fields below 50 kV/cm in order to ensure a consistent basis for comparison and to exclude extreme field conditions that may not be representative of practical device operation.

Several general trends emerge from these charts. Firstly, the most significant Δ*T* values, often exceeding 2 K, are consistently associated with darker colors, confirming that strong EC responses are typically observed only under applied fields of about 50 kV/cm [42], although some compositions also display relatively high Δ*T*/*E* ratios under more moderate fields (note that the Table A1 of the Appendix A includes additional compositions showing Δ*T* > 2 K; however, these values are obtained under very high electric fields, exceeding 100 kV/cm [27,32,69]). Secondly, the majority of responses cluster in the range of 300–380 K, close to the Curie temperature of BaTiO_3_, reinforcing that the ECE is maximized in the vicinity of the ferroelectric–paraelectric transition.

A-site substitutions usually yield moderate Δ*T* values of 0.5–1 K, with only a few outliers exceeding 2 K [34,42], and their responses are relatively broadly distributed in temperature. In contrast, B-site doping produces a broader spread of results, ranging from weak (<0.5 K) (e.g., [28,41,55,56,57,59,60,62,63]) up to strong (>2 K) (e.g., [40]) responses, indicating that B-site substitutions exert a stronger influence on polarization dynamics and phase stability. The highest responses, in some cases above 2.5 K [42,47], are recorded for co-doped compositions, underlining the synergistic benefits of simultaneously tuning both A- and B-sites.

**Figure 5:**
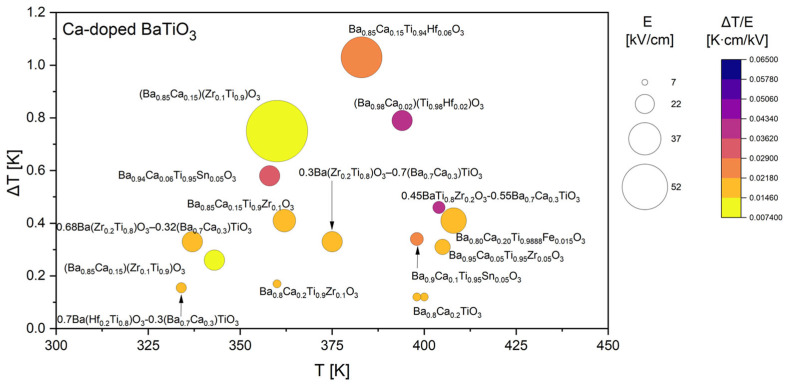
Electrocaloric response of Ca-doped BaTiO_3_: Δ*T* versus *T* [26,28,36,37,43,47,48,49,50,51,52,65,66,67,73]. Bubble size represents the applied electric field, while color indicates electrocaloric strength (Δ*T*/*E*). Only data obtained below 60 kV/cm are shown.

**Figure 6:**
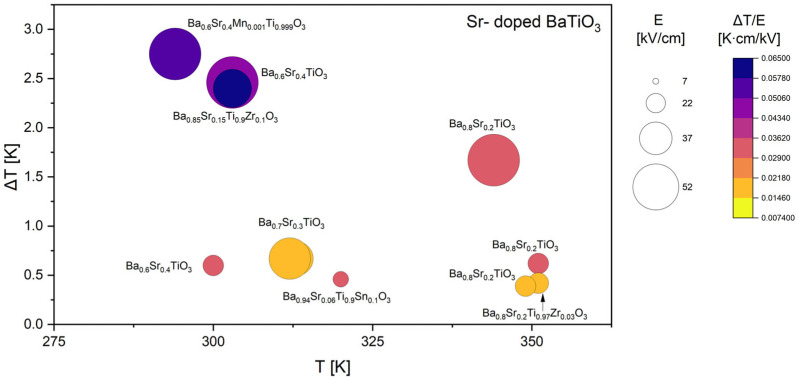
Electrocaloric response of Sr-doped BaTiO_3_: Δ*T* versus *T* [41,42,46,53,54,68,70,75]. Bubble size represents the applied electric field, while color indicates electrocaloric strength (Δ*T*/*E*). Only data obtained below 60 kV/cm are shown.

**Figure 7:**
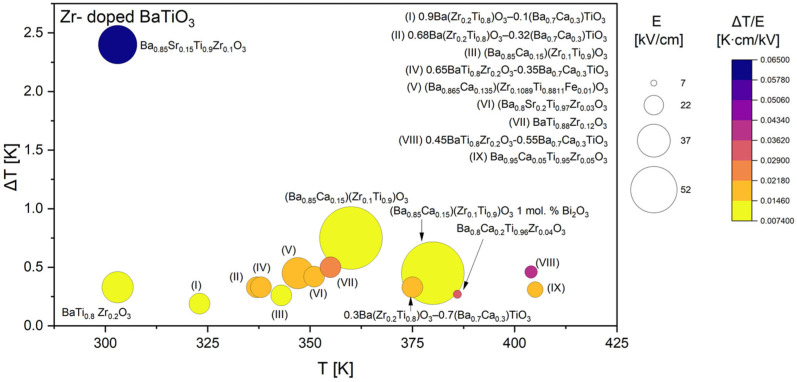
Electrocaloric response of Zr-doped BaTiO_3_: Δ*T* versus *T* [45,46,49,50,51,52,59,60,65,67,73,74]. Bubble size represents the applied electric field, while color indicates electrocaloric strength (Δ*T*/*E*). Only data obtained below 60 kV/cm are shown.

**Figure 8:**
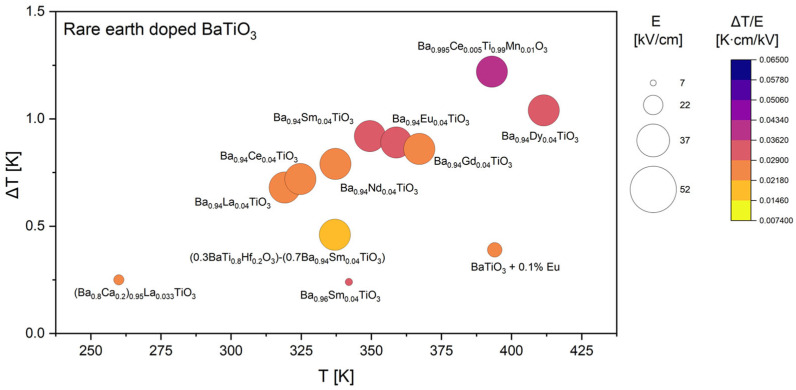
Electrocaloric response of rare earth-doped BaTiO_3_: Δ*T* versus *T* [21,22,24,29,36,38]. Bubble size represents the applied electric field, while color indicates electrocaloric strength (Δ*T*/*E*). Only data obtained below 60 kV/cm are shown.

Based on all collected data, the comparative analysis was subsequently divided into four focused groups, each discussed separately in the following sections. Since Ca, Sr, and Zr are among the most frequently used dopants in the literature, their datasets are presented and analyzed in greater detail (Figure 5, Figure 6 and Figure 7). It was also decided to discuss doping with rare-earth (Figure 8) elements, as they introduce local lattice distortions and polarization fluctuations through their unique ionic size and 4f electronic structure, enabling enhanced tunability and stabilization of the EC response. In cases where individual studies report complete compositional series, only the most representative results are included in the figures to ensure clarity and balanced comparison. In these detailed plots, the visualization is slightly different from that presented in Figure 4: the bubble size represents the applied electric field *E*, while the color scale corresponds to the EC strength (Δ*T*/*E*).

The EC performance of Ca-doped BaTiO_3_ presented in Figure 5 is strongly dependent on both the level of substitution and the presence of additional co-dopants such as Zr [47,49,50,51,67,73], Sn [26,66], Hf [28,37,48], and Fe [43]. Ca-modified compounds display a wide range of Δ*T* values, from below 0.1 K to nearly 1.0 K. This variability reflects the complex role of Ca^2+^ ions, which are smaller than Ba^2+^ and thus induce stronger lattice distortions while simultaneously shifting the ferroelectric transition temperature. The strongest responses are observed for multi-phase or co-doped systems rather than for simple Ca substitution. For example, Ba_0.85_Ca_0.15_Ti_0.94_Hf_0.06_O_3_ [37] and (Ba_0.85_Ca_0.15_)(Zr_0.1_Ti_0.9_)O_3_ [50] compositions reach Δ*T* values close to 1 K, highlighting the beneficial effect of simultaneously tuning the A- and B-sites. Similarly, Ba_0.94_Ca_0.06_Ti_0.95_Sn_0.05_O_3_ [66] shows an enhanced effect compared to simple Ca doping, again pointing to the role of co-substitution in broadening phase transitions and optimizing the entropy change. The large bubble sizes associated with these compositions indicate that such improvements are achieved under relatively high fields, but the corresponding Δ*T*/*E* values confirm that the intrinsic response is also significant.

A clear trend can be seen in the temperature dependence of the effect. The strongest EC responses cluster around 340–380 K, close to the Curie temperature of BaTiO_3_, which confirms that the mechanism is dominated by entropy changes near the ferroelectric–paraelectric phase boundary. At lower or higher temperatures, the effect is consistently weaker, as demonstrated by compositions such as Ba_0.8_Ca_0.2_TiO_3_ [36,65] or 0.7Ba(Hf_0.2_Ti_0.8_)O_3_–0.3(Ba_0.7_Ca_0.3_)TiO_3_ [48], both of which exhibit only minor Δ*T* values. This behavior again emphasizes that compositional tuning of the transition temperature is essential for maximizing EC strength.

Overall, the results demonstrate that Ca-doping in BaTiO_3_ provides significant compositional flexibility but also introduces sensitivity to stoichiometry and co-dopant choice. While simple Ca substitution yields only modest EC responses, carefully engineered multi-doped systems such as Ba_0.85_Ca_0.15_Ti_0.94_Hf_0.06_O_3_ [37] or Ba_0.94_Ca_0.06_Ti_0.95_Sn_0.05_O_3_ [66] show promising behavior, with Δ*T* values approaching 1 K near the ferroelectric transition. These findings highlight that the design of Ca-doped BaTiO_3_ electrocalorics should focus on balancing the destabilizing effect of Ca substitution with co-dopants that tune the transition temperature and enhance the entropy change.

Strontium substitution in BaTiO_3_ presented in Figure 6 leads to a markedly different EC behavior compared to Ca-doped systems. The most striking feature of the Sr-doped compositions is the exceptionally high Δ*T* values, in some cases exceeding 2.5 K, which are among the strongest responses reported for BaTiO_3_-based materials. In particular, Ba_0.6_Sr_0.4_TiO_3_ and Ba_0.6_Sr_0.4_Mn_0.001_Ti_0.999_O_3_ [42] exhibit large ECE in the temperature range around 290–300 K, with Δ*T*/*E* that are significantly higher than those observed for Ca-doped counterparts. The incorporation of Mn in small amounts further enhances the response, suggesting that subtle modifications of defect chemistry and domain wall pinning can play a synergistic role in maximizing the entropy change.

Compared to Ca substitution, where excessive doping suppresses ferroelectric order, Sr doping up to 40% appears to maintain strong polarization dynamics while shifting the ferroelectric transition temperature to lower values. This shift results in EC maxima appearing near room temperature, a particularly attractive feature for applications in solid-state cooling. The data also show that the ECE in Sr-doped BaTiO_3_ is less reliant on high electric fields compared to other dopant systems. Large Δ*T* values are obtained even under moderate field strengths, as indicated by the relatively high Δ*T*/*E* values in the purple region of the color scale. Note that if it were otherwise, the bubbles would be colored in the middle of the scale. It suggests that Sr doping effectively enhances the intrinsic EC coefficient by tuning the dielectric susceptibility near the transition. The results demonstrate that Sr substitution is highly effective in producing strong EC responses, with Δ*T* values exceeding 2 K in compositions with high Sr content at relatively low fields (below 50 kV/cm). The combination of large intrinsic Δ*T*/*E*, operation near room temperature, and reduced dependence on extreme electric fields makes Sr-doped BaTiO_3_ one of the most promising lead-free EC materials among the dopant families considered. The additional enhancement observed for Mn-modified Sr-doped BaTiO_3_ [42] further highlights the importance of co-doping strategies to fine-tune the interplay between lattice distortions, defect chemistry, and polarization dynamics.

Zirconium-modified BaTiO_3_ compositions presented in Figure 7 display a distinct EC behavior compared to Ca- or Sr-doped systems. In these materials, the amount of Zr incorporated into the lattice exerts a decisive influence on phase stability and the position of the ferroelectric transition. At low substitution levels (x < 0.1) [46,65,67], the EC temperature change remains comparable to that of undoped BaTiO_3_, typically in the range of 0.5–0.8 K [29,32,89]. With increasing Zr content, however, a systematic reduction in the maximum ΔT is observed, often falling below 0.3 K [50,52]. This decline can be attributed to the broadening of the ferroelectric–paraelectric transition and the partial suppression of long-range ferroelectric order, both of which diminish the entropy change associated with polarization switching. Despite the overall reduction in Δ*T*, compositions such as BaZr_0.12_Ti_0.88_O_3_ [60] or 0.45BaTi_0.8_Zr_0.2_O_3_–0.55Ba_0.7_Ca_0.3_TiO_3_ [73] exhibit relatively favorable Δ*T*/*E* values. That indicates that Zr substitution can enhance dielectric susceptibility and the intrinsic EC coefficient under certain conditions, even though the absolute temperature changes remain modest compared to those achieved in Sr-doped systems, where values exceeding 2 K are reported. On the other hand, the BaTi_1−x_Zr_x_O_3_ (BZT) solutions above the composition x = 0.2 exhibit relaxor behavior [32]. It is well known that the magnitude of Δ*T* in relaxors is highly competitive with that in normal ferroelectrics and the operational temperature window can be quite wide [32,74,101,102].

The temperature dependence of the effect further supports this interpretation: the EC maxima shift to lower temperatures with increasing Zr concentration, reflecting the downward movement of the Curie temperature, yet the overall magnitude of the response decreases simultaneously. The most favorable performance is typically observed around 320–350 K, where the modified transition still retains sufficient entropy change to drive a measurable effect. Taken together, these findings suggest that Zr doping in BaTiO_3_ is best regarded as a structural stabilizer rather than a direct enhancer of the EC response. While selected compositions can yield useful Δ*T*/*E* ratios [60,73], the maximum Δ*T* values are consistently lower than in Ca- or Sr-modified systems. As a result, Zr-doped BaTiO_3_ appears less promising for practical EC cooling applications, although it may play an important role in multiphase or co-doped systems where phase-boundary effects can be exploited.

The discussion concludes with an overview of rare-earth substituted BaTiO_3_ compositions. Looking at Figure 8 it can be seen that across the investigated compositions, the magnitude of the EC temperature change is typically on the order of 1 K, regardless of whether light rare-earth elements such as La [22,36], Ce [22,24], Nd [22], or Sm [21,22,38], or heavier elements such as Eu [22,29], Gd [22], and Dy [22] are employed. This observation suggests that the ECE is not strongly dependent on the atomic number of the rare-earth dopant itself, but rather on how the substitution perturbs the local lattice environment and the ferroelectric transition temperature.

A notable exception is the co-doped Ba_0.995_Ce_0.005_Ti_0.9_Mn_0.01_O_3_ [24] composition, which exhibits the highest Δ*T*/*E* in this dataset. The cooperative effect of Ce and Mn highlights the potential of co-doping strategies to optimize both phase stability and domain dynamics to maximize the caloric response. The role of dopant concentration is equally crucial. The strongest effects are consistently observed near a rare-earth substitution level of approximately four percent. Even small deviations from this composition, as demonstrated by the comparison between Ba_0.96_Sm_0.04_TiO_3_ [22] and Ba_0.94_Sm_0.04_TiO_3_ [38], can drastically reduce the ECE despite the application of comparable electric fields. This sensitivity indicates that the EC response is highly dependent on maintaining the delicate balance of lattice distortion and polar instability induced by the dopant ions.

Temperature dependence further reinforces the connection between the ECE and ferroelectric phase transitions. The majority of strong responses are concentrated in the range of 330–380 K, corresponding to the vicinity of the Curie temperature of BaTiO_3_. At lower temperatures, particularly in the case of Ca- and La-modified compositions [36] measured between 250 and 300 K, the magnitude of ΔT is significantly reduced.

Taken together, these results demonstrate that rare-earth doping in BaTiO_3_ provides a robust pathway to achieving ECEs on the order of 1 K, with the strongest enhancements arising from optimized dopant concentrations near the ferroelectric transition temperature. The pronounced response observed for the Ce–Mn co-doped system [24] suggests that rationally designed multi-element doping strategies may represent a promising route for further improvement of lead-free EC materials.

Although non-negligible, the electric field’s influence appears secondary to compositional and phase-related factors. Several compositions exhibit relatively strong ECEs under moderate applied fields, while others show weak responses even when subjected to higher fields. That underscores that tailoring the chemical composition is more effective than merely increasing the external stimulus.

The lack of standardized reporting practices in the ECE literature, particularly with regard to applied fields, sample microstructure, and measurement protocols, further limits comparability. We therefore strongly support the development of community-wide guidelines for reporting EC data, which would significantly facilitate both experimental cross-validation and machine-learning-based predictive modeling described in the next section.

### 3.2. Electrocaloric Effect Predictions from Machine Learning Models in the Literature

In recent years, the application of machine learning (ML) has emerged as a powerful strategy for predicting the ECE and accelerating the discovery of new ferroelectric materials. A number of recent studies [12,13,14,15] have established methodological frameworks that combine chemically informed descriptors, advanced regression models, and rigorous validation protocols. These approaches not only provide accurate predictive tools but also enable insights into the underlying physical mechanisms that govern polarization and entropy changes. Below, we present a detailed discussion of four representative contributions.

The predictive modeling of EC materials relies heavily on ML techniques capable of capturing complex, nonlinear relationships between chemical composition, structural features, and functional properties. Among the most commonly employed methods are decision trees and their ensemble extensions [12,13,15]. Decision trees are non-parametric models that recursively partition a dataset into subsets that are as homogeneous as possible with respect to the target property, such as adiabatic temperature change (Δ*T*) or isothermal entropy change (Δ*S*). Their transparent structure allows for the identification of key descriptors influencing the EC response.

While individual decision trees are easy to interpret [103], they may suffer from overfitting and limited predictive accuracy. To overcome these limitations, ensemble learning methods such as boosting are often applied [104]. Boosting algorithms iteratively combine multiple weak learners to form a stronger model. In Adaptive Boosting (AdaBoost), for instance, misclassified observations are given higher weight in successive iterations, improving the model’s ability to handle difficult-to-predict samples [105]. Gradient boosting, particularly its high-performance implementation XGBoost, optimizes an objective function using gradient descent in function space, offering both high accuracy and scalability [106]. These methods are especially effective in materials science, where datasets may include hundreds of descriptors derived from elemental properties, crystal structures, and processing conditions.

In the context of EC materials, ML workflows generally begin with the generation of chemically and physically informed descriptors, followed by feature selection procedures to remove redundant or irrelevant variables. This ensures robust and interpretable models that not only predict Δ*T* and Δ*S* with high accuracy but also highlight the material features most strongly correlated with EC performance [12]. The integration of decision-tree-based models and boosting algorithms has thus become a standard approach for accelerating the discovery and optimization of novel ferroelectric materials [13].

Gong et al. [12] developed one of the earliest large-scale ML frameworks for predicting ΔT in EC ceramics. Their workflow began with the systematic collection of data from literature, yielding 97 ceramic compositions and over 4000 datapoints. Each material was encoded using Magpie descriptors, which generate chemically informed features from elemental properties. To avoid redundancy, descriptors were filtered through variance analysis, Pearson correlation thresholds, and backward elimination.

The predictive model was built using eXtreme Gradient Boosting (XGBoost) regression, with hyperparameters optimized via an exhaustive grid search covering thousands of parameter combinations. The authors also implemented t-distributed stochastic neighbor embedding (t-SNE) and k-means clustering to guide the train–test split, ensuring that structurally distinct materials were fairly represented in both sets. Model development was carried out using Scikit-learn, TensorFlow, and PyTorch.

**Figure 9:**
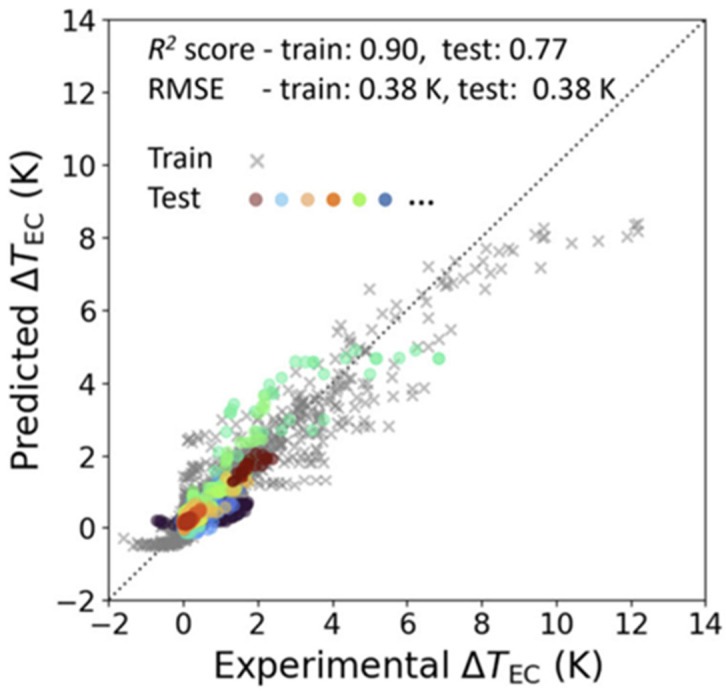
Parity plot of the predicted EC temperature change from one of the high-performing XGBoost models versus the experimental values. The gray crosses denote the training materials, the colored dots denote the test materials (different colors are used to distinguish different material compositions). Reprinted from Ref. [12].

Performance was assessed by comparing predicted and experimental Δ*T* values. The results (*R*^2^ ≈ 0.77, RMSE ≈ 0.38 K) showed close agreement, as illustrated in the parity plot Figure 9). Importantly, the model generalized beyond the training set, predicting unseen compositions with consistent accuracy.

**Figure 10:**
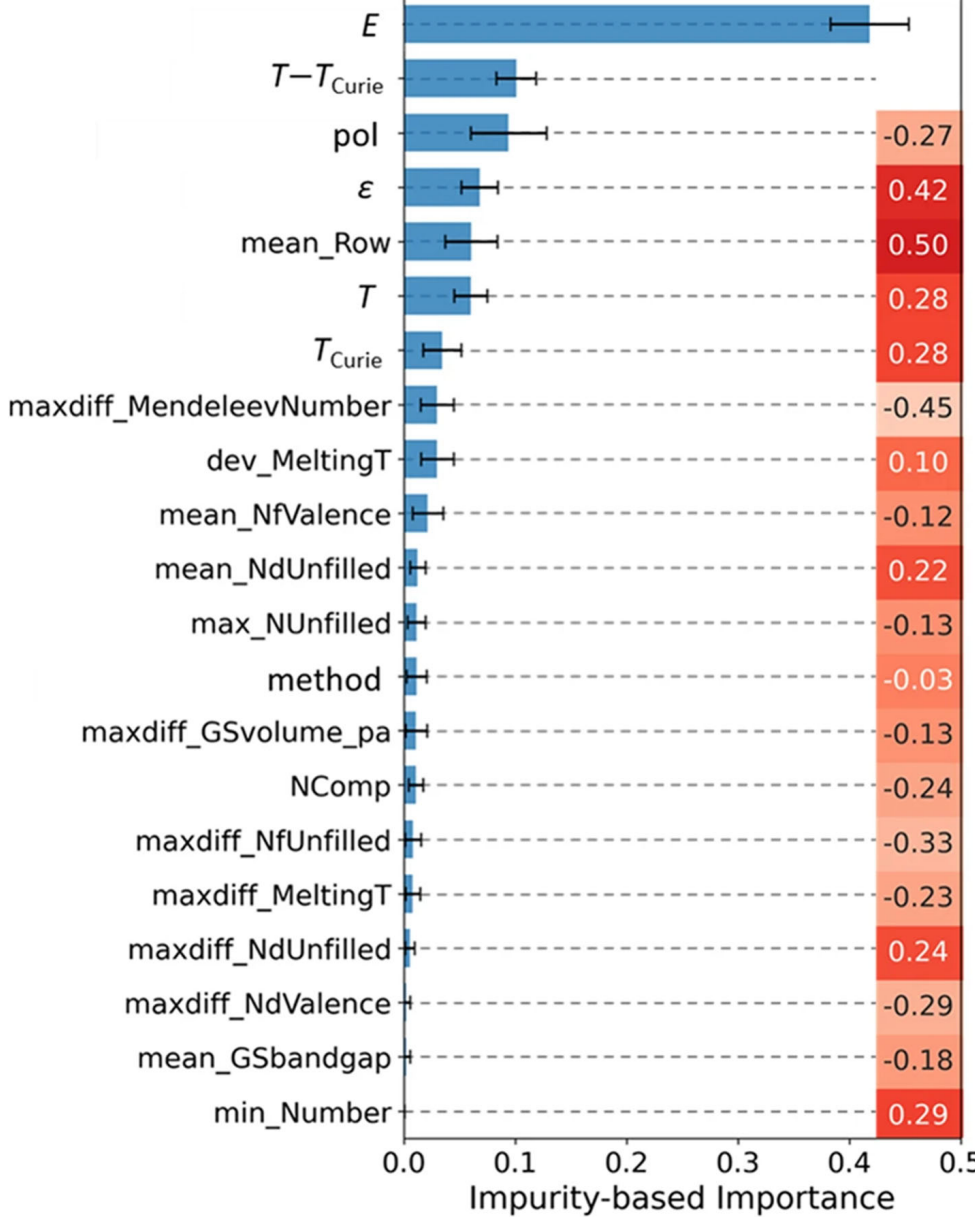
Impurity-based feature importance analysis. The error bar comes from the standard deviation of 100 random seeds. The color map shows the Pearson correlation coefficient of each feature with the predicted Δ*T*_EC_ in the model shown in Figure 9 after ruling out the influence of E and *T*-*T*_Curie_ by setting *E* = 100 kV/cm and *T* = *T*_Curie_. Reprinted from Ref. [12].

To gain physical insights, the authors analyzed feature importance using impurity-based metrics and correlation heatmaps. This revealed that electronegativity differences and ionic charge states were the dominant descriptors, directly linking the ML model to established ferroelectric theory, in which bond polarizability and charge asymmetry govern dipole reorientation. The feature importance plot (Figure 10) highlights these descriptors as the key drivers of Δ*T*.

**Figure 11:**
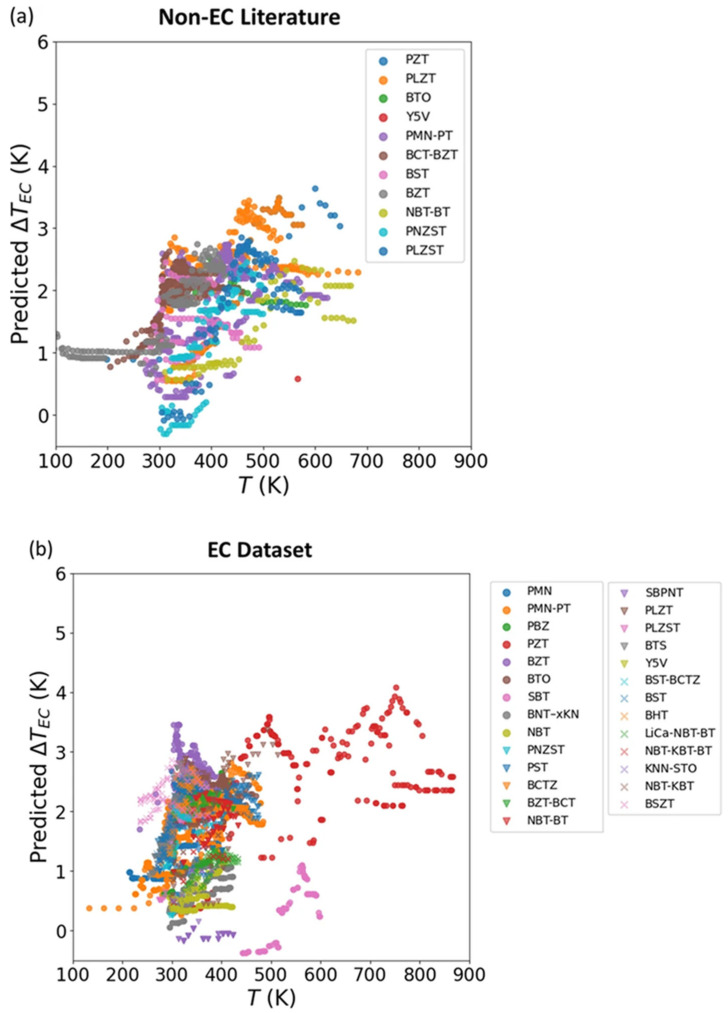
Predicted Δ*T*_EC_ at 100 kV/cm and varying temperature for ferroelectric ceramics. (**a**) predictions on the non-EC literature, (**b**) Predictions on the EC dataset. Reprinted from Ref. [12].

Finally, the model was extended to unexplored compositions, predicting Δ*T* values above 2 K under 100 kV cm^−1^ for several lead-free candidates at room temperature. This result suggests practical applicability for environmentally friendly cooling devices (Figure 11).

For illustration, we show here a comparison of the EC response for Ba_0.92_Ca_0.08_Ti_0.92_Zr_0.08_O_3_ with literature-reported experimental ECE data (Δ*T* = 0.38 K, *E* = 15 kV/cm, *T* = 409 K) [67], as well as for Ba_0.91_Ca_0.09_Ti_0.86_Zr_0.14_O_3_ with ML-predicted values (Δ*T* = 2.6 K, *E* = 100 kV/cm, *T* = 318 K) compiled by Gong et al. It should be emphasized that the properties of Ba_0.91_Ca_0.09_Ti_0.86_Zr_0.14_O_3_ have been reported in the literature [107], but no experimental EC values are available for it. Upon checking the Gong et al. database, we did not find evidence that the experimental values for Ba_0.92_Ca_0.08_Ti_0.92_Zr_0.08_O_3_ were included in the ML training dataset. Although slight differences in composition and measurement conditions should be considered, the comparison suggests that the ML predictions are reasonably representative and provide a meaningful benchmark against experimental trends. The observed difference in the temperature at which Δ*T* is measured can be attributed primarily to compositional effects, while larger applied electric effects increase the magnitude of Δ*T*. However, we do not aim to evaluate the complete accuracy of these values here.

Su et al. [13] focused specifically on BaTiO_3_-based ceramics, constructing a data-driven framework that distinguished between indirect and direct EC measurements. Two independent regression models were trained: (i) an SVR with Gaussian kernels and (ii) a random forest regression, supplemented by a support vector classification (SVC) model for predicting structural phase transitions.

Descriptors included ionic radii, electronegativity, tolerance factor, Mendeleev numbers, and dopant encoding. To reduce redundancy, pairwise correlation filtering was applied. Training was conducted in RStudio using the e1071 package, and ensembles were built via bootstrap resampling. Hyperparameters were tuned by grid search with cross-validation, ensuring robustness across multiple dataset partitions.

**Figure 12:**
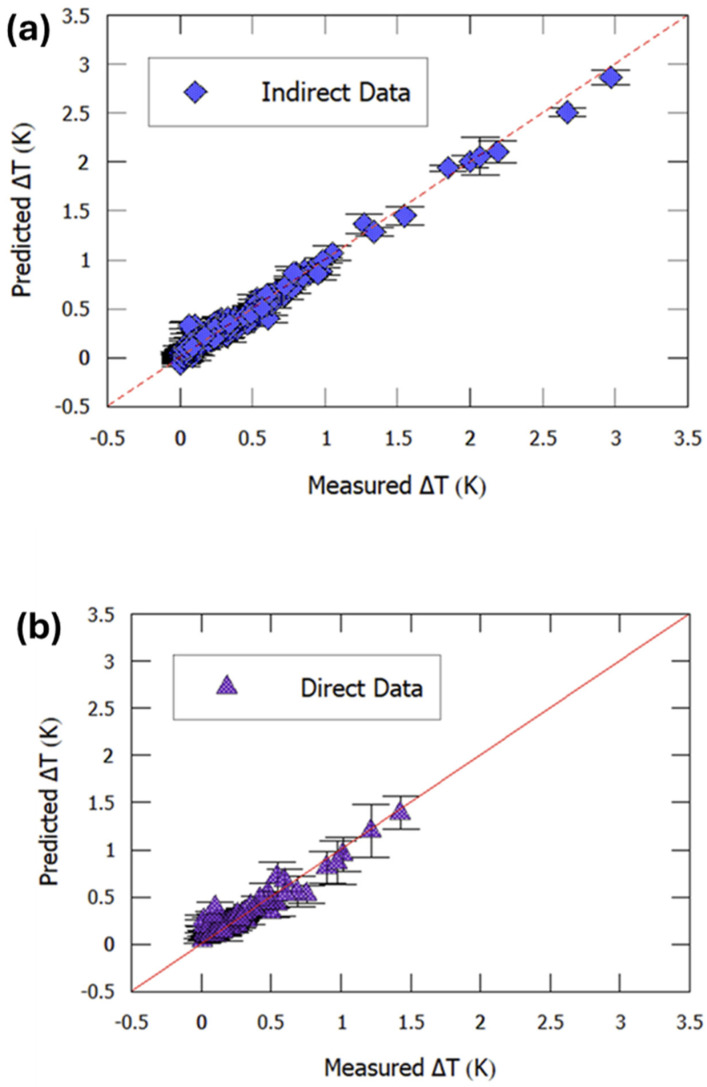
Performance plots of an ensemble of SVR models for (**a**) indirect and (**b**) direct data. The experimental Δ*T* is the *x*-axis and predictions from the trained models are on the *y*-axis. The error bars are the standard deviation of the predicted Δ*T*. All data points shown here correspond to those present in the independent test set, which the trained models have not seen before. (**a**) Indirect ensemble of SVR models with 20 bootstrap replications with the input descriptors: rTol_Fac, r_B, B_EN, D_EN, and T_K. (**b**) Direct ensemble of SVR models with 40 bootstrap replications with the input descriptors: rTol_Fac, r_B, B_EN, E and T_K. Reprinted with permission from Ref. [13]. Copyright 2021 American Chemical Society.

Model performance is shown in Figure 12. Both indirect and direct SVR ensembles reproduced experimental Δ*T* trends in unseen test sets, confirming that the models captured the nonlinear relationship between composition, structure, and EC response.

Beyond accuracy, Su et al. [13] also emphasized accessibility. They deployed their trained models in an interactive Shiny web application, allowing researchers to query predictions for new compositions in real time. From a physical perspective, the models highlighted that the tolerance factor and electronegativity differences were central to describing the EC response, consistent with long-standing concepts in perovskite ferroelectrics.

Nevertheless, the authors noted that dataset size and diversity remain limiting factors. They recommended systematic data sharing and automated literature extraction as future priorities to accelerate the adoption of informatics in EC research.

Bayir and Mensur [14] advanced the field by integrating XGBoost regression with Bayesian optimization for high-dimensional exploration of BCZT ceramics (Ba_x_Ca_1−x_Zr_γ_Ti_1−γ_O_3_). Their dataset comprised 2188 entries, including compositional ratios, processing parameters, and experimental conditions. Features combined Magpie descriptors (average atomic weight, electronegativity, atomic volume) with Curie temperature, explicitly encoding the role of phase transitions.

To ensure reliability, the dataset was split using group-aware cross-validation and ShuffleSplit, preventing data leakage from single publications. Bayesian optimization, implemented with the Optuna framework and the Tree-structured Parzen Estimator (TPE) algorithm, efficiently tuned hyperparameters while minimizing mean absolute error (MAE).

**Figure 13:**
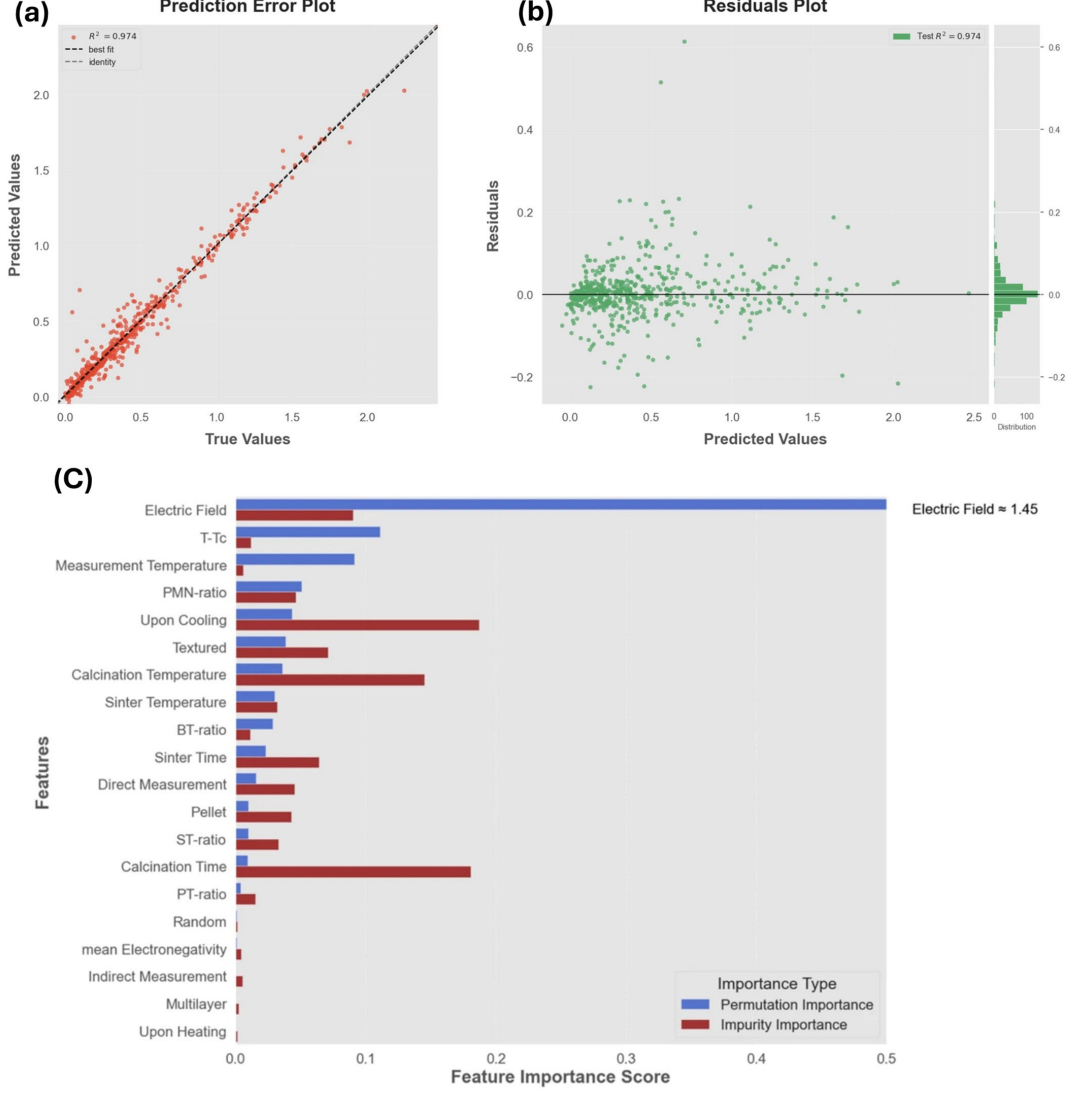
(**a**) Evaluation of XGBoost model performance for Δ*T*_EC_ prediction. Predicted versus true ΔT_EC_ values, (**b**) prediction residuals (difference between predicted and true values), (**c**) Feature importance analysis for Δ*T*_EC_ prediction using impurity-based ranking, SHAP values, and Pearson correlation. Reprinted from Ref. [14].

The resulting model achieved *R*^2^ ≈ 0.99 and MAE ≈ 0.02 °C, as shown in Figure 13, indicating nearly perfect predictive accuracy within the dataset. Feature importance analysis identified electric field strength, measurement temperature, and proximity to the Curie point as the most significant drivers of Δ*T*ₑc, echoing fundamental physical principles of entropy maximization near ferroelectric–paraelectric transitions.

Finally, Bayesian optimization was applied to identify optimal processing conditions, yielding a BCZT composition (Ba_0.85_Ca_0.15_Zr_0.4_Ti_0.6_) calcined at 1090 °C (3 h) and sintered at 1300 °C (2 h), predicted to achieve Δ*T*_e_c ≈ 1.03 °C at 24 °C under 40 kV cm^−1^. This showcases how ML not only reproduces physical trends but also directly informs materials design.

Last, Yuan et al. [15] proposed a surrogate modeling framework, leveraging polarization data as a proxy for EC properties. Instead of directly modeling Δ*T*, they constructed models predicting Curie temperature (*T*c) and polarization, then linked these to entropy change (Δ*S*) through a physics-informed surrogate.

**Figure 14:**
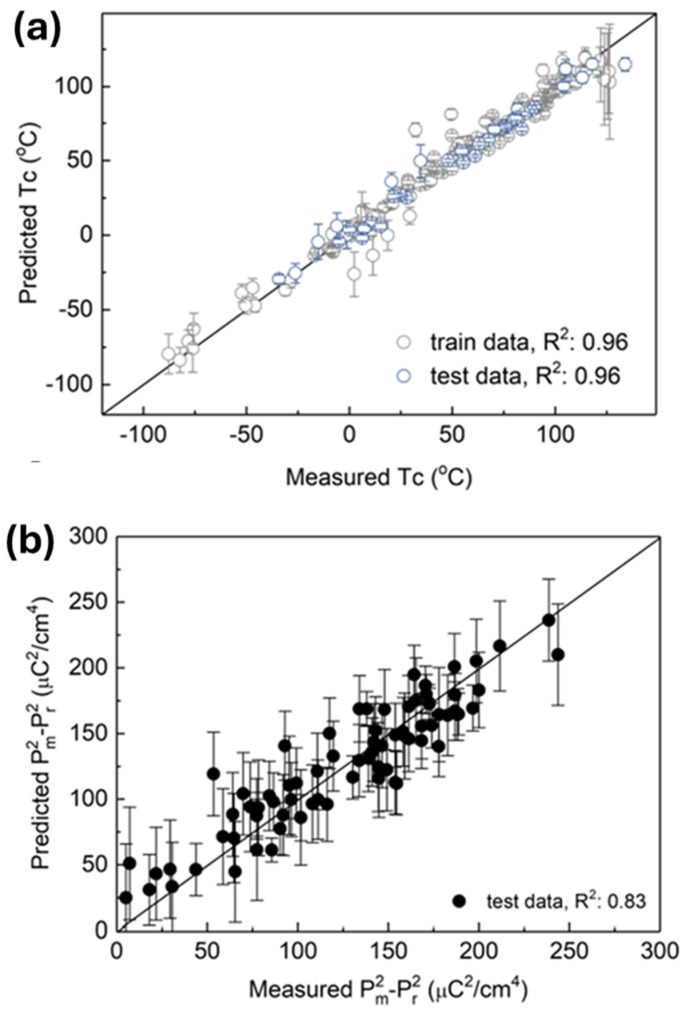
(**a**) The ML model for *T*c, showing the performance on the training data (gray points) as well as on the unseen test data (blue poins). (**b**) The ML model for polarization, comparing the model prediction to measurement. Data points in the test data are shown to validate the generalizability of ML model. Reprinted with permission from Ref. [15]. Copyright 2022 Elsevier.

Their dataset consisted of 195 BaTiO_3_-based compounds, characterized by five descriptors: NCT, D, tolerance factor, atomic volume, and electronegativity parameter (ENP). A support vector regression (SVR) with radial kernel was trained to predict *T*c, using 1000 bootstrap resamples with 10-fold CV to ensure robustness. The model reproduced *T*c with high accuracy, as shown in Figure 14.

Building on this, a Gaussian process regression (kriging-based, exponential kernel) was employed to model polarization behavior. Training began with 230 known compositions, after which the model was extended to 250,000 virtual compounds, enabling rapid high-throughput screening. Predictions aligned well with experimental polarization values (Figure 14b), validating the descriptors’ ability to capture dipole alignment mechanisms.

By coupling polarization predictions to a Landau-based thermodynamic model, the authors established a linear link between polarization and Δ*S* in moderate regimes, demonstrating that polarization can serve as a reliable surrogate for the ECE. This approach bridges the gap between data-driven methods and physical models, providing both predictive power and interpretability.

Across these four studies, a consistent methodological pattern emerges:

- Feature engineering: Magpie descriptors, ionic radii, tolerance factors, and polarization parameters;
- Dimensionality reduction and filtering: variance analysis, correlation thresholds, backward elimination;
- Models: ensemble regression (XGBoost, random forest, SVR), surrogate modeling (Gaussian process), and classification (SVC);
- Validation: bootstrap resampling, group-aware cross-validation, ShuffleSplit, and exhaustive grid/Bayesian optimization;
- Software frameworks: Scikit-learn, TensorFlow, PyTorch, RStudio (e1071), Optuna.

A structured overview of datasets, features, algorithms, validation strategies, and key results is provided in Table 2, which enables direct comparison across all four approaches.

## 4. Discussion

The analysis of the literature dataset confirms that BaTiO_3_-based ceramics remain the most widely studied family of lead-free EC materials, yet they also exemplify the intrinsic variability of reported results. Despite numerous experimental reports, values of the EC temperature change Δ*T* differ substantially between studies, even for nominally similar compositions. This scatter arises not only from intrinsic compositional effects but also from extrinsic factors such as grain size, porosity, electrode configuration, and above all, the measurement protocol employed. In particular, indirect results obtained via Maxwell’s relations may diverge significantly from direct calorimetric values. Such discrepancies highlight the necessity of cautious interpretation when comparing literature values.

Nevertheless, some robust qualitative patterns can be identified. Among A-site dopants, Sr substitution is particularly effective in enhancing the EC response, often shifting the peak Δ*T* toward room temperature and delivering values exceeding 2 K under moderate fields [42]. Ca-doping alone results in modest effects, but co-doping at both the A- and B-site (e.g., Ca–Hf or Ca–Sn systems [37,66]) enhances the response significantly. At the B-site, Zr substitution tends to suppress the maximum Δ*T* by broadening the ferroelectric transition and inducing relaxor behavior, although certain compositions exhibit favorable Δ*T*/*E* ratios [60,73]. It should be emphasized that although discrepancies between indirect and direct measurements are frequently observed, particularly in relaxor-type compositions, for certain solid solutions such as BZT–BCT [51], good agreement has been reported (0.318 K and 0.33 K for indirect and direct measurements, respectively, in the 0.68Ba(Zr_0.2_Ti_0.8_)O_3_–0.32(Ba_0.7_Ca_0.3_)TiO_3_). However, the inconsistency in other cases underscores that the reliability of indirect methods must be evaluated on a case-by-case basis, ideally by cross-checking against direct calorimetric techniques such as DSC.

Rare-earth substitutions typically produce responses of about 1 K, with optimized concentrations around 4% giving the best performance, and co-doping (e.g., Ce–Mn) further improving the effect [24]. These results demonstrate that while the absolute magnitude of Δ*T* remains variable, directional trends associated with specific dopant families are consistently observed across multiple independent studies.

The most critical weakness of the current body of work is the lack of full comparability between reported results. Literature data originate from different laboratories, using diverse synthesis routes, sintering conditions, and analysis procedures. This diversity introduces large systematic uncertainties and prevents truly quantitative benchmarking of EC performance across studies. Digitization of graphs, varying reporting standards, and incomplete descriptions of experimental conditions further exacerbate these issues. Consequently, while the identified trends are qualitatively meaningful, they cannot be considered precise or universal design rules.

Given these challenges, the establishment of standardized measurement and reporting practices is essential. Systematic documentation of applied electric fields, temperature ranges, grain size, density, and more would greatly enhance comparability across studies. Moreover, open repositories of raw polarization and calorimetry data, annotated with metadata, would provide a foundation for building benchmark datasets. Such standardized datasets are particularly critical for compositions like BaTiO_3_, Ba(Zr,Ti)O_3_–Ba(Ca,Ti)O_3_, and (Ba,Sr)TiO_3_, which could serve as community reference systems. Adopting these practices would significantly reduce uncertainty, facilitate meaningful cross-validation between direct and indirect results, and accelerate the development of predictive models.

In this context, machine learning (ML) has emerged as a promising but still early-stage tool for predicting EC behavior and accelerating materials discovery. The models developed so far successfully capture qualitative composition–property correlations across large, heterogeneous datasets, providing valuable guidance on dopant effects and substitutional trends. From a physical perspective, ML consistently identifies descriptors directly linked to ferroelectric mechanisms, such as electronegativity differences, ionic charge states, tolerance factor, and proximity to the Curie temperature [108,109]. These variables are central to dipole reorientation and entropy change, and their recurrence across different models reinforces the physical validity of the predictions. Notably, surrogate approaches linking polarization to entropy change have provided a bridge between data-driven methods and Landau-type thermodynamic theory [110].

From an informatics perspective, ensemble methods such as XGBoost and kernel-based regressors like SVR demonstrate strong predictive performance even on relatively small and heterogeneous datasets, particularly when combined with rigorous validation strategies such as bootstrap resampling, ShuffleSplit, or Bayesian optimization [12,13,14,15]. However, the predictive accuracy of ML is fundamentally limited by the scatter and inconsistency of the training data. Reported Δ*T* values vary not only with composition but also with field strength and measurement method, and ML models inevitably inherit this variability. As a result, current ML outcomes should be regarded as qualitative indicators rather than quantitative forecasts for specific synthesis routes. Moreover, most frameworks operate as black boxes, offering limited interpretability. Addressing this limitation requires the incorporation of physics-informed descriptors (already included in the models), such as tolerance factors or ionic radius variance, which indirectly capture structural distortions that drive ferroelectric transitions. Equally important is the inclusion of systematic uncertainty quantification through ensemble methods, Gaussian process regression, or other probabilistic approaches, so that predictions are accompanied by confidence intervals rather than single-point estimates.

It is important to situate ML within the broader computational landscape. Physics-based methods, including effective Hamiltonian models [111], first-principles (ab initio) simulations [112], and second-principles models (i.e., simplified, parametrized approaches derived from first-principles calculations) [113], offer complementary strengths. These methods capture intrinsic polarization dynamics, entropy changes, and phase-transition behavior with high physical interpretability, although they often involve significant computational cost and limited scalability. In contrast, ML approaches are data-driven, enabling rapid screening of large chemical spaces and excelling at uncovering empirical trends across diverse experimental datasets. To illustrate these complementary roles, Table 3 provides a comparative overview of their input requirements, strengths, limitations, and potential applications.

Looking forward, the reliable prediction and rational design of next-generation lead-free EC materials will require an integrated approach that combines standardized experimental protocols, uncertainty-aware ML models, and physics-informed mechanistic simulations. A next-generation ML framework could merge physics-informed surrogate models with advanced regressors, hybrid architectures, and transfer learning strategies, complemented by automated data augmentation (e.g., from high-throughput DFT or literature mining). Such a convergence of approaches would improve scalability, interpretability, and generalizability beyond BaTiO_3_-based systems, providing a robust platform to guide the discovery of new high-performance EC materials and bringing the field closer to practical solid-state cooling applications.

## 5. Conclusions

This work provided a comprehensive overview of the EC behavior of BaTiO_3_-based lead-free ceramics, focusing on the role of chemical substitution and the potential of emerging predictive approaches. The analysis of literature data confirmed several robust qualitative tendencies: the strongest EC responses occur close to the Curie temperature, typically within 300–380 K; Sr substitution produces particularly high Δ*T* values, often above 2 K under moderate fields, making it one of the most promising A-site strategies; Ca substitution alone yields modest responses, but co-doping at both the A- and B-sites (e.g., Ca-Hf or Ca-Sn) leads to clear improvements; Zr substitution broadens phase transitions and stabilizes the structure, often reducing the maximum Δ*T* while in some cases enhancing Δ*T*/*E*; and rare-earth dopants generally provide moderate responses around 1 K, with optimized concentrations near 4% and further enhancements in co-doped systems such as Ce-Mn.

At the same time, the compilation of results highlights that reported Δ*T* values for nominally similar compositions often differ significantly due to differences in sample preparation, microstructure, and measurement protocols. Such discrepancies underline the importance of careful interpretation and, wherever possible, cross-validation of results obtained with different experimental approaches.

Machine learning (ML) is emerging as a complementary tool for analyzing these heterogeneous data. Current models successfully capture qualitative composition- property correlations and consistently identify descriptors closely linked to ferroelectric mechanisms, providing useful guidance on substitution strategies. However, their predictive accuracy is limited by the variability and incompleteness of the available datasets, and ML outcomes should therefore be considered qualitative rather than quantitative. Incorporating physics-informed descriptors, uncertainty quantification, and improved standardization of reported experimental data will be key to enhancing the reliability of ML predictions. ML frameworks could also benefit from integration with insights obtained from first- and second-principles approaches, thereby combining rapid trend identification with mechanistic understanding.

In conclusion, meaningful insights into the EC behavior of BaTiO_3_-based ceramics can already be drawn from the literature, but further progress toward reliable prediction and practical optimization will depend on coordinated improvements in experimental standardization, data availability, and the integration of complementary predictive methods. Such convergence provides the most promising pathway for the rational design of next-generation lead-free EC materials, bringing environmentally friendly solid-state cooling technologies closer to application.

## Figures and Tables

**Table 1:** An example of the data organization. Symbols: Δ*T*—electrocaloric temperature change, *E*—applied electric field, Δ*T*/*E*—electrocaloric strength, I—indirect.

Composition	Δ*T* [K]	*E* [kV/cm]	*T* [K]	ΔT/E [K·cm/kV]	Method	Ref.
Ba_0.94_Sm_0.04_TiO_3_	0.920	30	350	0.0307	I	[22]
Ba_0.94_Dy_0.04_TiO_3_	1.040	30	412	0.0347	I	
Ba_0.94_La_0.04_TiO_3_	0.680	30	319	0.0227	I	
Ba_0.94_Ce_0.04_TiO_3_	0.720	30	325	0.0240	I	
Ba_0.94_Nd_0.04_TiO_3_	0.790	30	337	0.0263	I	
Ba_0.94_Eu_0.04_TiO_3_	0.890	30	359	0.0297	I	
Ba_0.94_Gd_0.04_TiO_3_	0.860	30	367	0.0287	I	

**Table 2:** Overview of machine learning approaches applied to electrocaloric materials in the literature.

Study	Dataset Size and Type	Features	ML Methods	Validation Strategy	Strengths	Limitations
Gong et al. [12]	~97 compositions, >4000 datapoints (literature)	Magpie descriptors, dielectric constant, Curie temperature, experimental conditions	XGBoost regression	t-SNE + k-means clustering, extensive grid search	R^2^ ≈ 0.77, RMSE ~0.38 K; captured physical trends (EN differences, ionic charge)	Dependent on heterogeneous literature data
Su et al. [13]	BaTiO_3_-based ceramics, indirect and direct data separated	Ionic radii, electronegativity, tolerance factor, dopant encoding	SVR (Gaussian kernels), Random Forest, ensemble models	Bootstrap resampling, grid search + cross-validation	Robust predictions for both direct and indirect datasets; interactive web app	Limited by dataset size and diversity
Bayir and Mensur [14]	2188 entries (BCZT ceramics, literature)	Magpie descriptors, Curie temperature, processing parameters	XGBoost + Bayesian optimization (Optuna TPE)	ShuffleSplit + group-aware cross-validation	R^2^ ≈ 0.99, MAE ≈ 0.02 °C; identified optimal BCZT composition	High accuracy but dataset-specific
Yuan et al. [15]	~195 compositions (BaTiO_3_-based)	Compositional descriptors (NCT, tolerance factor, atomic volume, EN, etc.)	SVR (radial kernel), Gaussian process surrogate models	Bootstrap (1000 samples) + 10-fold CV	Predicted Tc and polarization; surrogate model links polarization → ΔS	Requires indirect inference, not direct ΔT data

**Table 3:** Overview of physics-based methods and machine learning approaches for predicting the electrocaloric effect. MC—Monte Carlo, MD—Molecular Dynamics.

Method	Mechanism Captured	Typical Simulation Scale	Strengths	Limitations	Ref.
Effective Hamiltonian	Polar soft modes, coupling to strain; can include disorder if parameterized	10^3^–10^5^ unit cells, MC/MD	Mechanistic insight into ΔT, efficient for phase transitions, established for BTO/PTO, BZT	Requires careful DFT parametrization; difficult to include extrinsic defects; relaxors need large cells	[111]
Second principles	Anharmonic couplings, elasticity, mesoscale domains	10^5^–10^7^ atoms, longer MD	Captures domain/nanoregion effects; scalable to realistic microstructures	Parametrization heavy, limited transferability; still idealized vs. experiments	[113]
First principles MD	Full electronic structure, phonons, vibrational entropy	≤10^3^ atoms, short times	Highest fidelity per atom; captures electronic contributions	Too small/short to capture domains/disorder; very costly	[112]
Machine learning	Correlations between composition/process descriptors and ΔT	data-dependent/no single typical scale	Fast screening; finds statistical trends	Accuracy limited by data quality; limited mechanistics insights	[12,13,14,15]

## Data Availability

No new data were created or analyzed in this study. Data sharing is not applicable to this article.

## Appendix A

To provide a systematic overview of the reported EC properties of BaTiO_3_-based ceramics, the results collected from the literature are summarized in Table A1. The individual formulations have been reproduced exactly as presented in the original article, which employs varying notational conventions and may therefore appear somewhat inconsistent. The dataset includes both undoped and doped compositions, with key parameters described in Section 2.2. It should be emphasized that many of the values were obtained by digitizing published graphs rather than from tabulated data. As a result, small deviations relative to the original sources may occur, although every effort was made to preserve accuracy and reliability.

**Table A1:** BaTiO_3_ data from the literature. The individual formulations have been reproduced exactly as presented in the original article, which employs varying notational conventions. M denotes method, I—indirect, and D—direct.

Composition	Δ*T* [K]	*E* [kV/cm]	*T* [K]	Δ*T*/*E* [K∙cm/kV]	M	Ref.
BaTi_0.975_Y_0.025_O_3_	1.260	50.0	375	0.0252	I	[20]
BaTi_0.9625_Y_0.0375_O_3_	1.030	50.0	362	0.0206	I	
BaTi_0.95_Y_0.05_O_3_	1.150	50.0	345	0.0230	I	
BaTi_0.9375_Y_0.0625_O_3_	0.370	50.0	340	0.0074	I	
(0.6BaTi_0.8_Hf_0.2_O_3_)–(0.4Ba_0.94_Sm_0.04_TiO_3_)	0.340	30.0	334	0.0113	I	[21]
(0.5BaTi_0.8_Hf_0.2_O_3_)–(0.5Ba_0.94_Sm_0.04_TiO_3_)	0.390	30.0	341	0.0130	I	
(0.3BaTi_0.8_Hf_0.2_O_3_)–(0.7Ba_0.94_Sm_0.04_TiO_3_)	0.460	30.0	337	0.0153	I	
Ba_0.94_Sm_0.04_TiO_3_	0.920	30.0	350	0.0307	I	[22]
Ba_0.94_Dy_0.04_TiO_3_	1.040	30.0	412	0.0347	I	
Ba_0.94_La_0.04_TiO_3_	0.680	30.0	319	0.0227	I	
Ba_0.94_Ce_0.04_TiO_3_	0.720	30.0	325	0.0240	I	
Ba_0.94_Nd_0.04_TiO_3_	0.790	30.0	337	0.0263	I	
Ba_0.94_Eu_0.04_TiO_3_	0.890	30.0	359	0.0297	I	
Ba_0.94_Gd_0.04_TiO_3_	0.860	30.0	367	0.0287	I	
BaTiO_3_ + 4% Li	0.545	20.0	392	0.0273	I	[23]
BaTiO_3_ + 6% Li	0.575	20.0	393	0.0288	I	
BaTiO_3_ + 8% Li	0.525	20.0	393	0.0263	I	
Ba_0.995_Ce_0.005_Ti_0.99_Mn_0.01_O_3_	1.220	30.0	393	0.0407	I	[24]
Ba_0.985_Ce_0.015_Ti_0.99_Mn_0.01_O_3_	1.070	30.0	370	0.0357	I	
Ba_0.97_Ce_0.03_Ti_0.99_Mn_0.01_O_3_	0.410	30.0	328	0.0137	I	
Ba_0.96_Ce_0.04_Ti_0.99_Mn_0.01_O_3_	0.500	30.0	298	0.0167	I	
Ba_0.955_Ce_0.045_Ti_0.99_Mn_0.01_O_3_	0.420	30.0	293	0.0140	I	
BaTiO_3_	0.010	10.0	396	0.0010	I	[25]
BaTi_0.98_Sn_0.02_O_3_	0.160	9.8	370	0.0163	I	
BaTi_0.95_Sn_0.05_O_3_	0.079	3.6	343	0.0218	I	
BaTi_0.9_Sn_0.1_O_3_	0.050	10.1	306	0.0049	I	
Ba_0.98_Ca_0.02_Ti_0.95_Sn_0.05_O_3_	0.200	7.9	370	0.0253	I	[26]
Ba_0.95_Ca_0.05_Ti_0.95_Sn_0.05_O_3_	0.530	10.8	383	0.0490	I	
Ba_0.9_Ca_0.1_Ti_0.95_Sn_0.05_O_3_	0.340	12.7	398	0.0268	I	
0.8BaTi_0.82_Zr_0.18_O_3_–0.2BaTi_0.89_Sn_0.11_O_3_	3.500	100.0		0.0350	D	[27]
BaTi_0.82_Hf_0.18_O_3_	0.330	20.0	n/i	0.0165	I	[28]
BaTi_0.82_Hf_0.18_O_3_	0.370	20.0	n/i	0.0185	D	
(Ba_0.98_Ca_0.02_)(Ti_0.98_Hf_0.02_)O_3_	0.790	20.0	394	0.0395	I	
(Ba_0.87_Ca_0.13_)(Ti_0.87_Hf_0.13_)O_3_	0.470	20.0	328	0.0235	I	
(Ba_0.87_Ca_0.13_)(Ti_0.87_Hf_0.13_)O_3_	0.480	20.0	328	0.0240	D	
(Ba_0.86_Ca_0.14_)(Ti_0.86_Hf_0.14_)O_3_	0.350	20.0	324	0.0175	I	
(Ba_0.82_Ca_0.18_)(Ti_0.82_Hf_0.18_)O_3_	0.160	20.0	308	0.0080	I	
BaTiO_3_ + 0.1% Eu	0.390	14.0	394	0.0279	I	[29]
BaTiO_3_ + 1% Eu	0.300	10.0	387	0.0300	I	
BaTiO_3_ + 2% Eu	0.120	10.0	363	0.0120	I	
BaTiO_3_ + 3% Eu	0.070	10.0	391	0.0070	I	
BaTiO_3_	0.970	20.0	396	0.0485	I	[30]
BaTi_0.998_Nb_0.002_O_3_	0.820	20.0	396	0.0410	I	
BaTi_0.996_Nb_0.004_O_3_	0.790	20.0	392	0.0395	I	
BaTi_0.994_Nb_0.006_O_3_	0.660	20.0	385	0.0330	I	
BaTi_0.992_Nb_0.008_O_3_	0.500	20.0	384	0.0250	I	
BaTi_0.99_Nb_0.01_O_3_	0.440	20.0	n/i	0.0220	I	
BaTi_0.94_Sn_0.06_O_3_-Y	1.100	50.0	316	0.0220	I	[31]
BaTi_0.92_Sn_0.08_O_3_-Y	1.250	50.0	344	0.0250	I	
BaTi_0.89_Sn_0.11_O_3_-Y	1.400	50.0	328	0.0280	I	
BaTi_0.87_Sn_0.13_O_3_-Y	1.200	50.0	300	0.0240	I	
Ba(Zr_0.15_Ti_0.85_)O_3_	4.200	150.0	342	0.0280	D	[32]
Ba(Zr_0.2_Ti_0.8_)O_3_	4.500	145.0	312	0.0310	D	
(Ba_0.86_Ca_0.14_)_0.995_La_0.005_Ti_0.92_Sn_0.08_O_3_	0.850	40.0		0.0213	D	[33]
(Ba_0.86_Ca_0.14_)_0.99_La_0.01_Ti_0.92_Sn_0.08_O_3_	1.160	60.0		0.0193	D	
(Ba_0.86_Ca_0.14_)_0.985_La_0.015_Ti_0.92_Sn_0.08_O_3_	1.450	100.0		0.0145	D	
(Ba_0.86_Ca_0.14_)_0.98_La_0.02_Ti_0.92_Sn_0.08_O_3_	1.600	140.0		0.0114	D	
(Ba_0.86_Ca_0.14_)_0.975_La_0.025_Ti_0.92_Sn_0.08_O_3_	1.620	160.0		0.0101	D	
Ba_0.96_Pb_0.04_TiO_3_	2.190	18.0	420	0.1217	I	[34]
Ba_0.99_La_0.01_TiO_3_	0.600	60.0	420	0.0100	I	[35]
Ba_0.98_La_0.02_TiO_3_	0.650	60.0	370	0.0108	I	
Ba_0.97_La_0.03_TiO_3_	0.750	60.0	327	0.0125	I	
Ba_0.96_La_0.04_TiO_3_	1.810	60.0	303	0.0302	I	
(Ba_0.8_Ca_0.2_)TiO_3_	0.120	8.0	400	0.0151	I	[36]
(Ba_0.8_Ca_0.2_)_0.98_La_0.013_TiO_3_	0.115	9.9	370	0.0116	I	
(Ba_0.8_Ca_0.2_)_0.97_La_0.02_TiO_3_	0.150	9.9	310	0.0152	I	
(Ba_0.8_Ca_0.2_)_0.95_La_0.033_TiO_3_	0.250	9.9	260	0.0253	I	
Ba_0.85_Ca_0.15_Ti_0.94_Hf_0.06_O_3_	1.030	40.0	383	0.0258	D	[37]
Ba_0.85_Ca_0.15_Ti_0.9_Hf_0.1_O_3_	0.800	40.0	373	0.0200	D	
Ba_0.85_Ca_0.15_Ti_0.85_Hf_0.15_O_3_	0.540	40.0	333	0.0135	D	
Ba_0.96_Sm_0.04_TiO_3_	0.240	7.0	342	0.0343	D	[38]
Ba_0.7_Sr_0.3_TiO_3_ + 1mol% Br^+^ + 0.3 mol% Mn^2+^	0.650	20.0	313	0.0325	I	[39]
Ba_0.7_Sr_0.3_TiO_3_ + 1mol% Br^+^ + 0.5 mol% Mn^2+^	0.860	20.0	311	0.0430	I	
Ba_0.7_Sr_0.3_TiO_3_ + 1mol% Br^+^ + 1.5 mol% Mn^2+^	0.400	20.0	308	0.0200	I	
BaTi_0.9_Hf_0.1_O_3_	1.990	50.0	353	0.0398	D	[40]
0.2BaTi_0.93_ Sn_0.07_O_3_–0.8BaTi_0.9_Hf_0.1_O_3_	2.160	50.0	353	0.0432	D	
BaTi_0.93_Sn_0.07_O_3_	1.140	50.0	353	0.0228	D	
BaTi_0.9_Sn_0.1_O_3_	0.480	15.0	337	0.0320	I	[41]
Ba_0.94_Sr_0.06_Ti_0.9_Sn_0.1_O_3_	0.460	15.0	320	0.0307	I	
Ba_0.93_Sr_0.07_Ti_0.9_Sn_0.1_O_3_	0.370	15.0	315	0.0247	I	
Ba_0.92_Sr_0.08_Ti_0.9_Sn_0.1_O_3_	0.340	15.0	304	0.0227	I	
Ba_0.6_Sr_0.4_TiO_3_	2.460	50.0	303	0.0492	D	[42]
Ba_0.6_Sr_0.4_Mn_0.001_Ti_0.999_O_3_	2.750	50.0	294	0.0550	D	
Ba_0.6_Sr_0.4_Mn_0.002_Ti_0.998_O_3_	2.570	50.0	283	0.0514	D	
Ba_0.6_Sr_0.4_Mn_0.003_Ti_0.997_O_3_	2.450	50.0	273	0.0490	D	
Ba_0.6_Sr_0.4_Mn_0.004_Ti_0.996_O_3_	2.290	50.0	263	0.0458	D	
Ba_0.6_Sr_0.4_Mn_0.005_Ti_0.995_O_3_	2.090	50.0	243	0.0418	D	
Ba_0.65_Sr_0.35_TiO_3_	2.420	50.0	283	0.0484	D	
Ba_0.65_Sr_0.35_Mn_0.001_Ti_0.999_O_3_	2.580	50.0	263	0.0516	D	
Ba_0.65_Sr_0.35_Mn_0.002_Ti_0.998_O_3_	2.340	50.0	263	0.0468	D	
Ba_0.65_Sr_0.35_Mn_0.003_Ti_0.997_O_3_	2.280	50.0	263	0.0456	D	
Ba_0.65_Sr_0.35_Mn_0.004_Ti_0.996_O_3_	2.260	50.0	281	0.0452	D	
Ba_0.65_Sr_0.35_Mn_0.005_Ti_0.995_O_3_	1.980	50.0	243	0.0396	D	
Ba_0.80_Ca_0.20_Ti_0.9925_Fe_0.01_O_3_	0.370	25.0	396	0.0148	I	[43]
Ba_0.80_Ca_0.20_Ti_0.9888_Fe_0.015_O_3_	0.410	25.0	408	0.0164	I	
Ba_0.80_Ca_0.20_Ti_0.985_Fe_0.02_O_3_	0.440	25.0	368	0.0176	I	
Ba(Ge_0.02_Ti_0.98_)O_3_	0.550	8.0	400	0.0692	I	[44]
Ba(Ge_0.03_Ti_0.97_)O_3_	0.590	8.0	401	0.0742	I	
Ba(Ge_0.05_Ti_0.95_)O_3_	0.530	8.0	402	0.0667	I	
Ba(Ge_0.06_Ti_0.94_)O_3_	0.810	8.0	403	0.1019	I	
Ba(Ge_0.09_Ti_0.91_)O_3_	0.380	8.0	404	0.0478	I	
Ba(Ge_0.06_Ti_0.94_)O_3_	0.904	8.0	400	0.1130	D	
(Ba_0.865_Ca_0.135_)(Zr_0.1089_Ti_0.8811_Fe_0.01_)O_3_	0.450	30.0	347	0.0150	I	[45]
Ba_0.8_Sr_0.2_TiO_3_	0.620	20.0	351	0.0310	I	[46]
Ba_0.8_Sr_0.2_Ti_0.97_Zr_0.03_O_3_	0.420	20.0	351	0.0210	I	
Ba_0.8_Sr_0.2_Ti_0.95_Zr_0.05_O_3_	0.430	20.0	351	0.0215	I	
Ba_0.8_Sr_0.2_Ti_0.93_Zr_0.07_O_3_	0.400	20.0	351	0.0200	I	
Ba_0.8_Sr_0.2_Ti_0.9_Zr_0.1_O_3_	0.350	20.0	342	0.0175	I	
Ba_0.85_Ca_0.15_Ti_0.9_Zr_0.1_O_3_	0.410	22.0	362	0.0186	I	[47]
Ba_0.85_Ca_0.1_Sr_0.05_Ti_0.9_Zr_0.1_O_3_	0.600	30.0	333	0.0200	I	
Ba_0.85_Ca_0.05_Sr_0.1_Ti_0.9_Zr_0.1_O_3_	1.000	30.0	323	0.0333	I	
Ba_0.85_Sr_0.15_Ti_0.9_Zr_0.1_O_3_	2.400	37.0	303	0.0649	I	
0.8Ba(Hf_0.2_Ti_0.8_)O_3_-0.2(Ba_0.7_Ca_0.3_)TiO_3_	0.130	10.0	324	0.0130	I	[48]
0.7Ba(Hf_0.2_Ti_0.8_)O_3_-0.3(Ba_0.7_Ca_0.3_)TiO_3_	0.155	10.0	334	0.0155	I	
0.6Ba(Hf_0.2_Ti_0.8_)O_3_-0.4(Ba_0.7_Ca_0.3_)TiO_3_	0.140	10.0	352	0.0140	I	
0.5Ba(Hf_0.2_Ti_0.8_)O_3_-0.5(Ba_0.7_Ca_0.3_)TiO_3_	0.240	10.0	362	0.0240	D	
0.8Ba(Hf_0.2_Ti_0.8_)O_3_-0.2(Ba_0.7_Ca_0.3_)TiO_3_	0.125	10.0	313	0.0125	D	
0.7Ba(Hf_0.2_Ti_0.8_)O_3_-0.3(Ba_0.7_Ca_0.3_)TiO_3_	0.200	10.0	333	0.0200	D	
0.6Ba(Hf_0.2_Ti_0.8_)O_3_-0.4(Ba_0.7_Ca_0.3_)TiO_3_	0.150	10.0	343	0.0150	D	
0.5Ba(Hf_0.2_Ti_0.8_)O_3_-0.5(Ba_0.7_Ca_0.3_)TiO_3_	0.270	10.0	363	0.0270		
(Ba_0.85_Ca_0.15_)(Zr_0.1_Ti_0.9_)O_3_	0.750	60.0	360	0.0125	D	[49]
(Ba_0.85_Ca_0.15_)(Zr_0.1_Ti_0.9_)O_3_ 1 mol. % Bi_2_O_3_	0.450	60.0	380	0.0075	D	
(Ba_0.85_Ca_0.15_)(Zr_0.1_Ti_0.9_)O_3_	0.260	20.0	343	0.0130	I	[50]
(Ba_0.85_Ca_0.15_)(Zr_0.1_Ti_0.9_)O_3_	0.464	60.0	373	0.0077	D	
0.68Ba(Zr_0.2_Ti_0.8_)O_3_–0.32(Ba_0.7_Ca_0.3_)TiO_3_	0.318	20.0	352	0.0159	I	[51]
0.65Ba(Zr_0.2_Ti_0.8_)O_3_–0.35(Ba_0.7_Ca_0.3_)TiO_3_	0.188	20.0	348	0.0094	I	
0.63Ba(Zr_0.2_Ti_0.8_)O_3_–0.37(Ba_0.7_Ca_0.3_)TiO_3_	0.155	20.0	359	0.0078	I	
0.6Ba(Zr_0.2_Ti_0.8_)O_3_–0.4(Ba_0.7_Ca_0.3_)TiO_3_	0.225	20.0	361	0.0113	I	
0.55Ba(Zr_0.2_Ti_0.8_)O_3_–0.45(Ba_0.7_Ca_0.3_)TiO_3_	0.205	20.0	363	0.0103	I	
0.68Ba(Zr0.2Ti0.8)O3–0.32(Ba0.7Ca0.3)TiO3	0.330	20.0	337	0.0165	D	
0.65Ba(Zr_0.2_Ti_0.8_)O_3_–0.35(Ba_0.7_Ca_0.3_)TiO_3_	0.250	20.0	338	0.0125	D	
0.63Ba(Zr_0.2_Ti_0.8_)O_3_–0.37(Ba_0.7_Ca_0.3_)TiO_3_	0.195	20.0	343	0.0098	D	
0.6Ba(Zr_0.2_Ti_0.8_)O_3_–0.4(Ba_0.7_Ca_0.3_)TiO_3_	0.230	20.0	348	0.0115	D	
0.55Ba(Zr_0.2_Ti_0.8_)O_3_–0.45(Ba_0.7_Ca_0.3_)TiO_3_	0.255	20.0	362	0.0128	D	
0.9Ba(Zr_0.2_Ti_0.8_)O_3_–0.1(Ba_0.7_Ca_0.3_)TiO_3_	0.190	20.0	323	0.0095	I	[52]
0.7Ba(Zr_0.2_Ti_0.8_)O_3_–0.3(Ba_0.7_Ca_0.3_)TiO_3_	0.300	20.0	330	0.0150	I	
0.65Ba(Zr_0.2_Ti_0.8_)O_3_–0.35(Ba_0.7_Ca_0.3_)TiO_3_	0.280	20.0	348	0.0140	I	
0.5 0.5Ba(Zr_0.2_Ti_0.8_)O_3_–0.5(Ba_0.7_Ca_0.3_)TiO_3_	0.290	20.0	363	0.0145	I	
0.3Ba(Zr_0.2_Ti_0.8_)O_3_–0.7(Ba_0.7_Ca_0.3_)TiO_3_	0.330	20.0	375	0.0165	I	
Ba_0.6_Sr_0.4_TiO_3_	0.600	20.0	300	0.0300	D	[53]
Ba_0.7_Sr_0.3_TiO_3_	0.670	33.0	313	0.0203	I	[54]
Ba_0.8_Sr_0.2_TiO_3_	0.830	33.0	338	0.0252	I	
Ba_0.9_Sr_0.1_TiO_3_	0.610	33.0	360	0.0185	I	
BaTi_0.99_In_0.01_O_2.995_	0.220	10.0	290	0.0220	I	[55]
BaTi_0.97_In_0.03_O_2.985_	0.290	26.0	324	0.0112	I	
BaTi_0.95_In_0.05_O_2.975_	0.420	26.0	314	0.0162	I	
BaTi_0.944_Y_0.056_O_2.972_	0.400	45.0	356	0.0089	I	[56]
BaTi_0.942_Y_0.058_O_2.971_	0.375	45.0	344	0.0083	I	
BaTi_0.94_Y_0.06_O_2.97_	0.340	45.0	338	0.0076	I	
BaTi_0.938_Y_0.062_O_2.969_	0.340	45.0	338	0.0076	I	
BaTi_0.936_Y_0.064_O_2.968_	0.330	45.0	338	0.0073	I	
BaTi_0.97_Hf_0.03_O_3_	0.190	10.0	318	0.0190	D	[57]
BaTi_0.89_Hf_0.11_O_3_	0.350	10.0	343	0.0350	D	
BaTi_0.83_Hf_0.17_O_3_	0.320	10.0	313	0.0320	D	
BaTiO_3_	0.925	24.0	396	0.0385	I	[58]
BaTi_0.99_Ce_0.1_O_3_	0.450	24.0	371	0.0188	I	
BaTi_0.88_Ce_0.12_O_3_	0.825	24.0	351	0.0344	I	
BaTi_0.85_Ce_0.15_O_3_	0.650	24.0	338	0.0271	I	
BaTi_0.8_ Zr_0.2_O_3_	0.330	30.0	303	0.0110	I	[59]
BaTi_0.88_Zr_0.12_O_3_	0.500	20.0	355	0.0250	D	[60]
BaTi_0.8_Zr_0.2_O_3_	0.300	20.0	314	0.0150	D	
0.05 BaTi_0.95_Sn_0.05_O_3_	1.450	30.0	368	0.0483	D	[61]
0.1BaTi_0.9_Sn_0.1_O_3_	1.240	30.0	338	0.0413	D	
BaTi_0.85_Sn_0.15_O_3_	1.490	30.0	299	0.0497	D	
BaTi_0.9_Sn_0.1_O_3_	0.400	20.0	343	0.0200	I	[62]
BaTi_0.88_Sn_0.12_O_3_	0.450	20.0	325	0.0225	I	
BaTi_0.85_Sn_0.15_O_3_	0.420	20.0	301	0.0210	I	
BaTi_0.82_Sn_0.18_O_3_	0.318	20.0	298	0.0159	I	
BaTi_0.92_Sn_0.08_O_3_	0.490	20.0	341	0.0245	D	[63]
BaTi_0.89_Sn_0.11_O_3_	0.630	20.0	317	0.0315	D	
BaTi_0.86_Sn_0.14_O_3_	0.390	20.0	291	0.0195	D	
BaTi_0.85_Sn_0.15_O_3_	0.310	20.0	289	0.0155	D	
BaTi_0.92_Sn_0.08_O_3_	0.520	20.0	331	0.0260	I	[64]
BaTi_0.895_Sn_0.105_O_3_	0.610	20.0	301	0.0305	I	
BaTi_0.86_Sn_0.14_O_3_	0.470	20.0	282	0.0235	I	
Ba_0.8_Ca_0.2_TiO_3_	0.120	8.0	398	0.0151	I	[65]
Ba_0.8_Ca_0.2_Ti_0.96_Zr_0.04_O_3_	0.270	8.0	386	0.0340	I	
Ba_0.8_Ca_0.2_Ti_0.9_Zr_0.1_O_3_	0.170	8.0	360	0.0214	I	
Ba_0.94_Ca_0.06_Ti_0.95_Sn_0.05_O_3_	0.580	20.0	358	0.0290	I	[66]
Ba_0.94_Ca_0.06_Ti_0.925_Sn_0.075_O_3_	0.580	20.0	339	0.0290	I	
Ba_0.94_Ca_0.06_Ti_0.9_Sn_0.1_O_3_	0.550	20.0	320	0.0275	I	
Ba_0.94_Ca_0.06_Ti_0.875_Sn_0.125_O_3_	0.630	20.0	298	0.0315	I	
Ba_0.94_Ca_0.06_Ti_0.85_Sn_0.15_O_3_	0.350	20.0	281	0.0175	I	
Ba_0.94_Ca_0.06_Ti_0.8_Sn_0.2_O_3_	0.320	20.0	237	0.0160	I	
Ba_0.95_Ca_0.05_Ti_0.95_Zr_0.05_O_3_	0.310	15.0	405	0.0207	I	[67]
Ba_0.92_Ca_0.08_Ti_0.92_Zr_0.08_O_3_	0.380	15.0	409	0.0253	I	
Ba_0.9_Ca_0.1_Ti_0.92_Zr_0.08_O_3_	0.230	15.0	406	0.0153	I	
Ba_0.8_Sr_0.2_TiO_3_	0.390	20.0	349	0.0195	I	[68]
Ba_0.75_Sr_0.25_TiO_3_	0.370	20.0	334	0.0185	I	
Ba_0.7_Sr_0.3_TiO_3_	0.360	20.0	303	0.0180	I	
Ba_0.65_Sr_0.35_TiO_3_	0.420	20.0	296	0.0210	I	
Ba_0.8_Zr_0.2_TiO_3_	2.780	161.0	350	0.0173	I	[69]
Ba_0.7_Sr_0.3_TiO_3_ con	0.670	40.0	312	0.0168	I	[70]
Ba_0.7_Sr_0.3_TiO_3_ SPS	1.850	90.0	312	0.0206	I	
Ba_0.7_Sr_0.3_Ti_0.997_Mn_0.003_O_3_ SPS	2.530	120.0	303	0.0211	I	
Bi_0.5_Na_0.5_TiO_3_–0.06BaTiO_3_	0.860	50.0	383	0.0172	D	[71]
Ba_0.95_Ca_0.05_Ti_0.94_Sn_0.06_O_3_	1.600	180.0	300	0.0089	I	[72]
0.45BaTi_0.8_Zr_0.2_O_3_-0.55Ba_0.7_Ca_0.3_TiO_3_	0.460	12.0	404	0.0383	I	[73]
0.65BaTi_0.8_Zr_0.2_O_3_-0.35Ba_0.7_Ca_0.3_TiO_3_	0.330	20.0	338	0.0165	D	[74]
Ba_0.8_Sr_0.2_TiO_3_	1.670	50.0	344	0.0334	I	[75]
Ba_0.65_Sr_0.35_TiO_3_ Con	0.830	90.0	303	0.0092	I	[76]
Ba_0.65_Sr_0.35_TiO_3_ SPS	2.100	90.0	303	0.0233	I	
Ba_0.65_Sr_0.35_Ti_0.997_Mn_0.003_O_3_ SPS	3.080	130.0	293	0.0237	I	
